# Intra- and interannual dynamics of grassland community phylogenetic structure are influenced by meteorological conditions before the growing season

**DOI:** 10.3389/fpls.2022.870526

**Published:** 2022-09-23

**Authors:** Lei Dong, Ying Zheng, Jian Wang, Jinrong Li, Zhiyong Li, Jinghui Zhang, Lixin Wang, Bailing Miao, Cunzhu Liang

**Affiliations:** ^1^ Yinshanbeilu Grassland Eco-hydrology National Observation and Research Station, China Institute of Water Resources and Hydropower Research, Beijing, China; ^2^ Institute of Water Resources for Pastoral Areas, Ministry of Water Resources, Hohhot, China; ^3^ School of Ecology and Environment, Inner Mongolia University, Hohhot, China; ^4^ Meteorological Research Institute of Inner Mongolia, Inner Mongolia Meteorological Service, Hohhot, China

**Keywords:** community phylogenetic structure, extreme climate, Inner Mongolia, interspecies competition, intra-annual dynamics, net relatedness index, steppe

## Abstract

The impact of global climate change on ecosystem structure has attracted much attention from researchers. However, how climate change and meteorological conditions influence community phylogenetic structure remains poorly understood. In this research, we quantified the responses of grassland communities’ phylogenetic structure to long- and short-term meteorological conditions in Inner Mongolia, China. The net relatedness index (NRI) was used to characterize phylogenetic structure, and the relationship between the NRI and climate data was analyzed to understand the dynamics of community phylogenetic structure and its relationship with extreme meteorological events. Furthermore, multiple linear regression and structural equation models (SEMs) were used to quantify the relative contributions of meteorological factors before and during the current growing season to short-term changes in community phylogenetic structure. In addition, we evaluated the effect of long-term meteorological factors on yearly NRI anomalies with classification and regression trees (CARTs). We found that 1) the degree of phylogenetic clustering of the community is relatively low in the peak growing season, when habitat filtering is relatively weak and competition is fiercer. 2) Extreme meteorological conditions (i.e., drought and cold) may change community phylogenetic structure and indirectly reduce the degree of phylogenetic clustering by reducing the proportion of dominant perennial grasses. 3) Meteorological conditions before the growing season rather than during the current growing season explain more variation in the NRI and interannual NRI anomalies. Our results may provide useful information for understanding grassland community species assembly and how climate change affects biodiversity.

## Introduction

Global climate change has deeply influenced biodiversity and community structure (Willis et al., 2008). Therefore, knowing how communities and ecosystems respond to climate change is urgently needed. Herbs respond more quickly to environmental changes than trees and shrubs (Ren et al., 2020); therefore, herbs are an ideal object for studying the impact of climate change on ecosystems. Temporal dynamics in community biomass (Briggs and Knapp, 1995; Tilman et al., 2001; Yang et al., 2010), biodiversity (Tilman et al., 2006; Fargione et al., 2007), functional groups (Voigt et al., 2007; Griffin-Nolan et al., 2019), and their relationships with the climate (Bai et al., 2004; Bernhardt‐Römermann et al., 2011) have attracted much attention from researchers. Previous studies have confirmed that different species respond differently to climate conditions (VanderWeide and Hartnett, 2015), so changes in climate conditions can also change community species composition. For instance, Evans et al. (2011) found that drought significantly decreased dominant perennial grass while ruderals increased. Clark et al. (2002) also found a decrease in perennial grass and an increase in annuals and forbs in grasslands under drought in the northern Great Plains, USA. Wang et al. (2012) showed that warming significantly increased perennial grass but reduced non-legume forbs. Some researchers found that previous-year precipitation had a significant influence on current-year community (Oesterheld et al., 2001; Carter et al., 2012).

However, most researchers draw their conclusion based on data from the peak growing season, and seasonal phenology of community species coexistence has been overlooked (Munson and Lauenroth, 2009). In natural ecosystems, species tend to avoid competition by using shared resources at different times (i.e., temporal niche separation) (Schofield et al., 2018; Doležal et al., 2019), thereby a seasonal dynamic of species composition is expected. For instance, studies in North American shortgrass prairie showed that C3 grasses and forbs predominated early in the growing season, but by the mid-growing season, the C4 grass *Bouteloua gracilis* became the dominant species in the community (Oesterheld et al., 2001; Griffin-Nolan et al., 2019). Therefore, the species composition and interspecific relationship in the community may also change at different stages of the growing season.

With the availability of molecular phylogenies, analysis of community structure is now possible at the phylogenetic level rather than the species level (Webb et al., 2002). Generally, closely related species tend to be ecologically similar, and the interaction between evolutionary relatedness of coexistence species and biotic and abiotic factors may lead to non-random patterns of phylogenetic structure among constituent species (i.e., community phylogenetic structure; see Swenson et al., 2007). Community phylogenetic structure could provide a new perspective on how climate change influences community structure and species assembly (Lavergne et al., 2010). Previous studies have focused on either the long-term successional dynamics (i.e., Kembel and Hubbell, 2006; Döbert et al., 2017; Yu et al., 2019) or the local/regional average status (i.e., Graham et al., 2009; Chi et al., 2014; Zhang et al., 2020) of community phylogenetic structure. These studies treat community phylogenetic structure as relatively stable on a short time scale (e.g., seasonal scale). On the other hand, the temporal dynamics of communities (e.g., biomass and species richness), as other researchers have proven (i.e., Tilman et al., 2006; Yang et al., 2010), are closely related to the dynamics of community phylogenetic structure. However, the seasonal and yearly dynamics of community phylogenetic structure remain unclear.

Recently, some researchers have noticed that migration may change local community composition. For example, Che et al. (2019) reported the intra-annual dynamics of waterbird community phylogenetic and functional structures in a subtropical wetland under the influence of migratory bird migration. Specifically, waterbird assemblages in migration seasons but not in non-migration seasons are significantly phylogenetically clustered, whereas they are phylogenetically overdispersed in winter. Ramírez et al. (2015) showed that bee community phylogenetic structure changes in response to seasonal rainfall fluctuations. Other researchers reported the seasonal dynamic of microbial (i.e., Martin and Hans-Peter, 2006; Andersson et al., 2010; Sun et al., 2021) and parasitic (i.e., Luo et al., 2010; Wang et al., 2021) community phylogenetic structure and/or phylogenetic diversity; however, research on plant community composition mainly remains at the species or functional group level (Oesterheld et al., 2001; Thomey et al., 2011; Griffin-Nolan et al., 2019), and the knowledge about the temporal dynamics of plant community phylogenetic structure remains unclear. Notably, how community phylogenetic structure responds to long- and short-term meteorological and climate change is still poorly understood.

In this research, we examined the intra- and interannual dynamics of the community phylogenetic structure of two typical steppe grasslands and their relationships with meteorological factors. We hypothesized that 1) the intra-annual community phylogenetic structures would differ depending on changes in the growing season and meteorology, i.e., habitat filtering would play a greater role in community assembly at the beginning of the growing season than in the peak growing season. Therefore, the degree of clustering should be greater (i.e., taxa are more phylogenetically similar than expected) than that in the peak growing season. 2) Community phylogenetic structure would show obvious interannual dynamics in response to changes in meteorological conditions, and the responses by community phylogenetic structure to meteorological conditions would have a time lag.

## Materials and methods

### Site description

The study was conducted in Xilinhot, Inner Mongolia, China. The climate is a semiarid temperate continental climate, and nearly 70% of precipitation occurs from May to September. The vegetation type is typical steppe, and the soil is *Kastanozem* according to the WRB (Dong et al., 2021). The growing season starts at the end of April and ends in late September.

**Figure 1:**
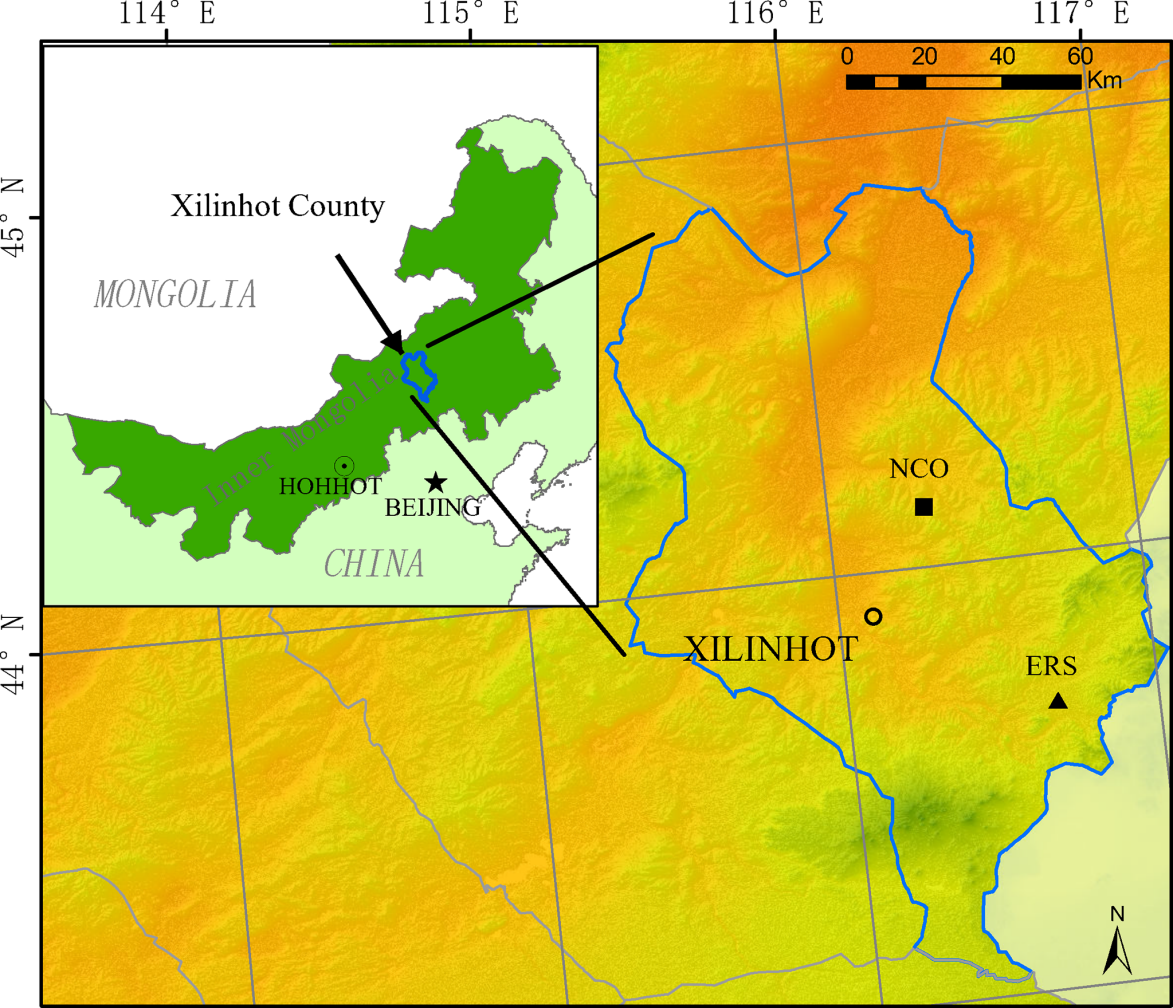
Location of the two experimental sites (ERS, Inner Mongolia Grassland Ecosystem Research Station; NCO, Xilinhot National Climate Observatory).

In this study, we analyzed data collected from two different grassland habitats in Xilinhot (**Figure 1**). The first site is located at the Inner Mongolia Grassland Ecosystem Research Station (IMGERS, 43°33′N, 116°40′E), approximately 70 km southeast of Xilinhot (site ERS hereafter). The altitude is approximately 1,190 m above sea level. The mean annual temperature and precipitation are 1.1°C and 331.4 mm, respectively (1982–2020). In 1979, a fence (24 ha, 600 m × 400 m) was constructed, and since then, all grazing has been excluded. Before fence construction, the grassland was severely degraded due to heavy grazing. After decades of restoration, the vegetation has been significantly restored. The dominant species are *Leymus chinensis* and *Stipa grandis*.

The second site is located at the Xilinhot National Climate Observatory (44°08′N, 116°20′), approximately 15 km east of Xilinhot (site NCO hereafter). The altitude is 1,129 m above sea level. The mean annual temperature and precipitation are 3.2°C and 273.7 mm, respectively (1982–2020). The climate at NCO is drier and the species richness is lower than that at ERS. NCO has free grazing with low intensity before the experiment began, and the dominant species is *S. grandis.* In 2012, three 1.44-ha (120 m × 120 m) fences were constructed to prevent grazing (more details are provided by Dong et al., 2021).

### Quadrat sampling

Sampling at ERS was conducted once every half month in the growing season. Sampling began on May 15 and ended on September 15, so nine samplings were performed each year. For each sampling, 20 adjacent 1 m × 1 m quadrats were set and surveyed. Sampling at NCO began on May 20, and samples were taken on the 10th, 20th, and 30th of each month until September 20. That is, a total of 13 samplings were performed each year. For each sampling, 15 1 m × 1 m quadrats (3 fences × 5 randomly established quadrats) were set and surveyed.

In each quadrat, all the species were recorded, and for each species, living aboveground tissue was clipped from individual plants and stored in a paper bag. Plant materials were dried in an oven (at 75°C, approximately 20 h), after which the weights were determined.

In this research, we used two databases. The first database is a short-term database of quadrat data collected from ERS and NCO for seven consecutive years (from 2014 to 2020). It is worth noting that at NCO, data are not available for 30 August 2018, 20 May 2020, and 20 June 2020. The second database is a long-term database for site ERS from 1984 to 2020 (except in 1995 and 1996, i.e., 35 years). The long-term database contains only data from two periods in the peak growing season in each year (i.e., sampling on August 15 and 30).

### Meteorological data

Meteorological data from ERS and NCO were obtained from the weather stations of IMGERS (http://www.cnern.org.cn) and the China Meteorological Data Service Centre (http://data.cma.cn), respectively. We considered 2017 to be a drought year because it was the second driest year in the past 15 years. Precipitation at ERS and NCO was equivalent to only 84.05% (278.2 mm) and 61.31% (168.6 mm) of the annual average precipitation, respectively (**Figure S1**). We chose eight meteorological indices to analyze the impact of meteorological conditions on community structure. These eight indicators were classified into two categories. The first category included four past meteorological indicators (i.e., those before the growing season), namely the average temperature of the last coldest month (i.e., January, *T*_Jan_), extremely low temperature in January (*T*_min_), precipitation of the past years (*P*_past_), and precipitation of the years with less precipitation in the last 2 years (*P*_less2_). The second category included four current meteorological indicators (i.e., those of the current growing season), namely the average temperature of the last 15 days before sampling (*T*_15_), current accumulated temperature >0°C (from 1 January until the sampling day, CAT0), accumulated precipitation of the last 15 days before sampling (*P*_15_), and current total accumulated precipitation (from 1 January until the sampling day, CAP).

### Phylogenetic structure analysis

We built a phylogenetic tree using all vascular plants that were recorded at both sites. The phylogenetic tree was constructed in R package V.PhyloMaker (Jin and Qian, 2019). V.PhyloMaker uses GBOTB (i.e., GenBank taxa with a backbone provided by Open Tree of Life) as a backbone to generate a phylogeny (Smith and Brown, 2018). GBOTB includes all accepted families of vascular plants (according to APG IV) in the world. The species that were absent from the mega-tree were bound to the halfway point of the family (or genus) branch (by setting “Scenario = S3” in function *phylo.maker*) (Jin and Qian, 2019; Qian et al., 2019).

We used the net relatedness index (NRI), weighted by relative dry aboveground biomass of individual species (i.e., set “abundance.weighted = TRUE”), as an index of community phylogenetic structure. Relative dry aboveground biomass (RDAB) of species *i* was calculated as follows:


$$RDBA_{i}\;=\;\frac{dry\;aboveground\;biomass\;of\;species\;i}{dry\;aboveground\;biomass\;of\;quadrat\;}$$

The NRI was calculated as follows:


$$NRI\;=\;-1\;\times \;\frac{MPD_{obs}\;-\;MPD_{null}}{sd\;(MPD_{null})}$$

where NRI is the net relatedness index, MPD_obs_ is the observed mean phylogenetic distance (MPD) in the quadrat, MPD_null_ are 999 random MPD values under the null model, and sd (MPD_null_) is the standard deviation of MPD_null_ (Webb et al., 2002). The NRI was calculated in package “picante” (NRI is equivalent to −1 times the output of “ses.mpd”; see Kembel et al., 2010). A positive NRI indicates phylogenetic clustering (i.e., taxa are more phylogenetically similar than expected by chance), while a negative NRI indicates phylogenetic overdispersion (i.e., taxa are more phylogenetically distant than expected by chance) (Swenson et al., 2006).

### Statistical analysis

All statistical analyses were performed using R 3.5.3 (R CoreTeam, 2019). We used one-way ANOVA followed by Duncan’s multiple range test (significance level *p* < 0.05) (De Mendiburu, 2014) to determine the significance of intra- and interannual differences in the NRI. If the NRI in the peak growing season was significantly lower than at the beginning and end of the growing season, habitat filtering would play a greater role in community assembly at the beginning and end of the growing season than in the peak growing season and, thus, lead to the intra-annual dynamic of the phylogenetic structure. We used multiple linear regression and structural equation models (SEMs) to quantify the relative contributions of meteorological factors before and during the current growing season to short-term community phylogenetic structure at both sites. The best multiple linear regression model was identified as that with the minimum Akaike information criterion (AIC). The SEM was fitted by maximum likelihood estimation using the “lavaan” package (Rosseel, 2012). An acceptable SEM fit was defined as a non-significant *χ* ^2^ test result (*p* > 0.05), high goodness-of-fit index (GFI > 0.95), low standardized root mean square residual (SRMSR < 0.08), and low root mean square error of approximation (RMSEA < 0.05) (Schermelleh-Engel et al., 2003).

We also used yearly NRI anomalies (i.e., NRI of the last year – NRI of the current year, NRI_an_) to reflect the influence of meteorological factors on community phylogenetic structure under long time series. A negative NRI_an_ indicates a declining NRI when compared to that of the last year, and a positive NRI_an_ indicates an increasing NRI when compared to that of the last year. We evaluated the effects of meteorological factors on NRI_an_ with classification and regression trees (CARTs) using the “rpart” package (Therneau et al., 2015). To avoid overfitting, the complexity parameter (cp) was set according to the smallest cross-validation error (xerror). In addition, we used linear mixed-effects models (LMMs) to evaluate the relationships between meteorological factors and NRI_an_, incorporating potential meteorological factors as fixed effects and year as a random intercept. The LMMs were fitted using the “lme4” package (Bates et al., 2014), and the variance explained by the LMMs was calculated using the “MuMIn” package (Barton, 2020).

## Results

### Community phylogenetic structure

**Figure 2:**
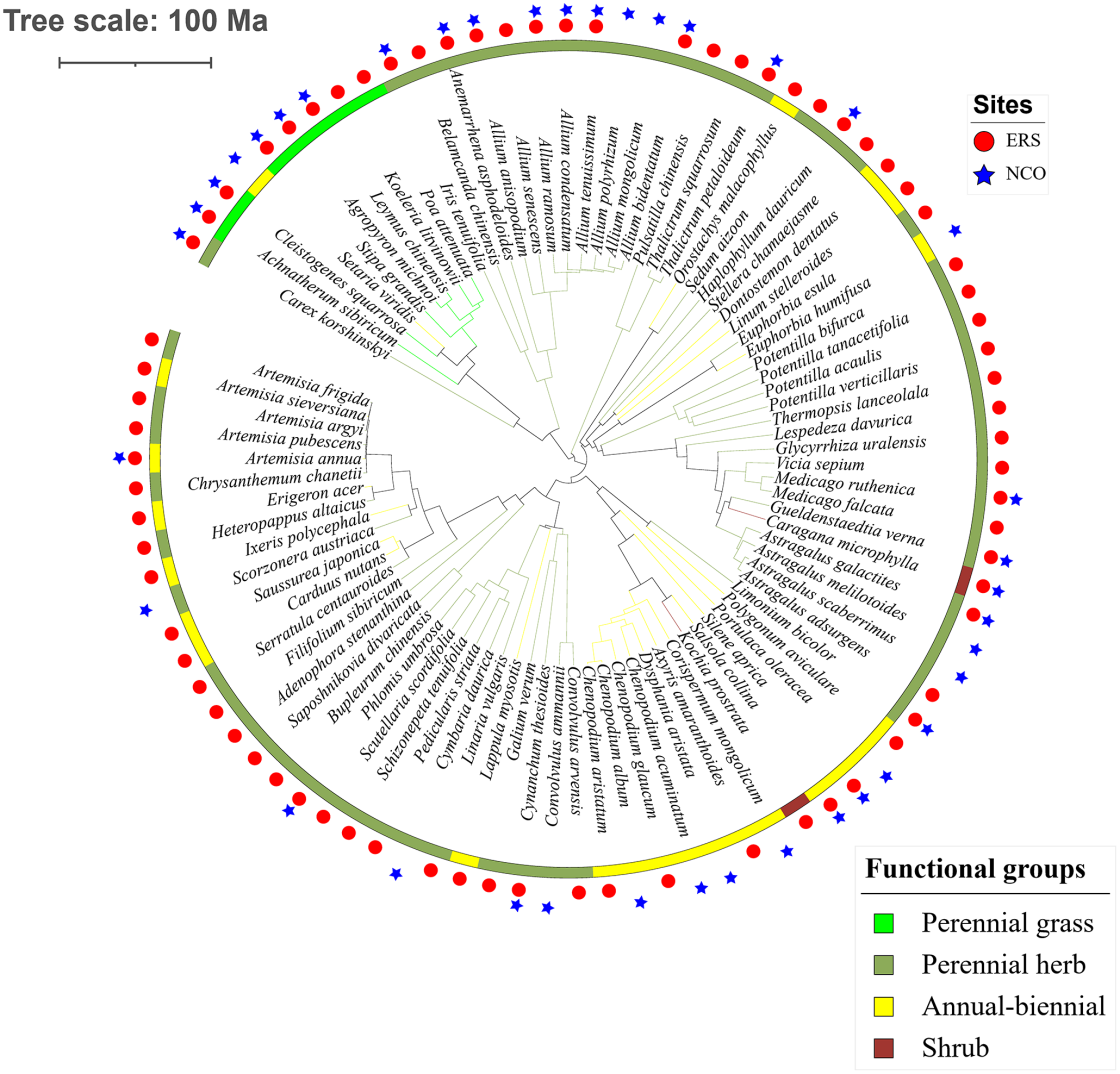
Phylogenetic tree of the 89 species observed in this study. Circles and stars represent species recorded at Inner Mongolia Grassland Ecosystem Research Station (ERS) and Xilinhot National Climate Observatory (NCO) in Inner Mongolia, China, respectively.

**Figure 3:**
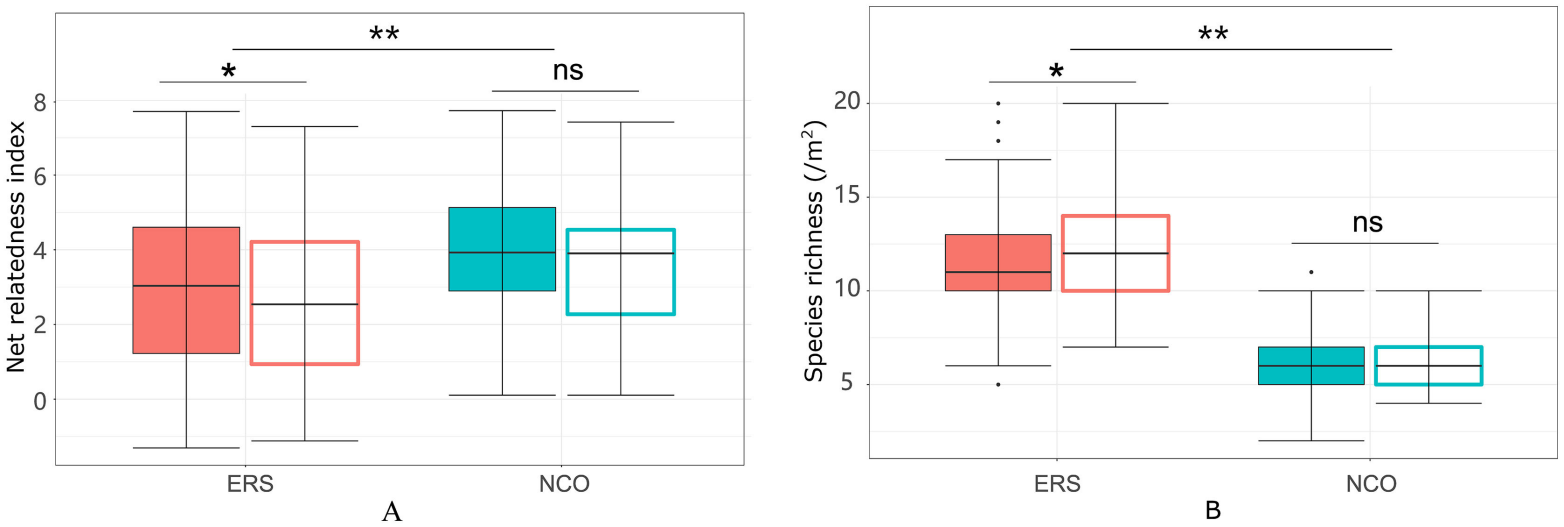
Net relatedness index **(A)** and species richness **(B)** at the Inner Mongolia Grassland Ecosystem Research Station (ERS) and Xilinhot National Climate Observatory (NCO) in Inner Mongolia, China. Filled boxes indicate annual mean NRIs (or species richness), and empty boxes indicate peak growing season NRIs (or species richness). A single asterisk and double asterisk indicate *p <*0.05 and *p <*0.01, respectively, according to Duncan’s *post hoc* test. ns, not significant.

Eighty-eight species belonging to 29 families and 64 genera were recorded in this research. The species richness at ERS was significantly higher than that at NCO (75 species at ERS and 39 species at NCO, respectively, **Figure 2** and **Figure S2**). In addition, the NRI was significantly greater than 0 at both sites, indicating phylogenetic clustering. The average NRI at ERS was significantly lower than that at NCO. We also calculated the average NRI and species richness in the peak growing season (i.e., in late August). We found that at site ERS, in the peak growing season, the NRI and species richness were significantly lower and higher than the annual mean values, respectively. In contrast, the difference in the NRI and species richness between the peak growing season and annual mean at the NCO site was not significant (**Figure 3**).

### Intra-annual dynamics

**Figure 4:**
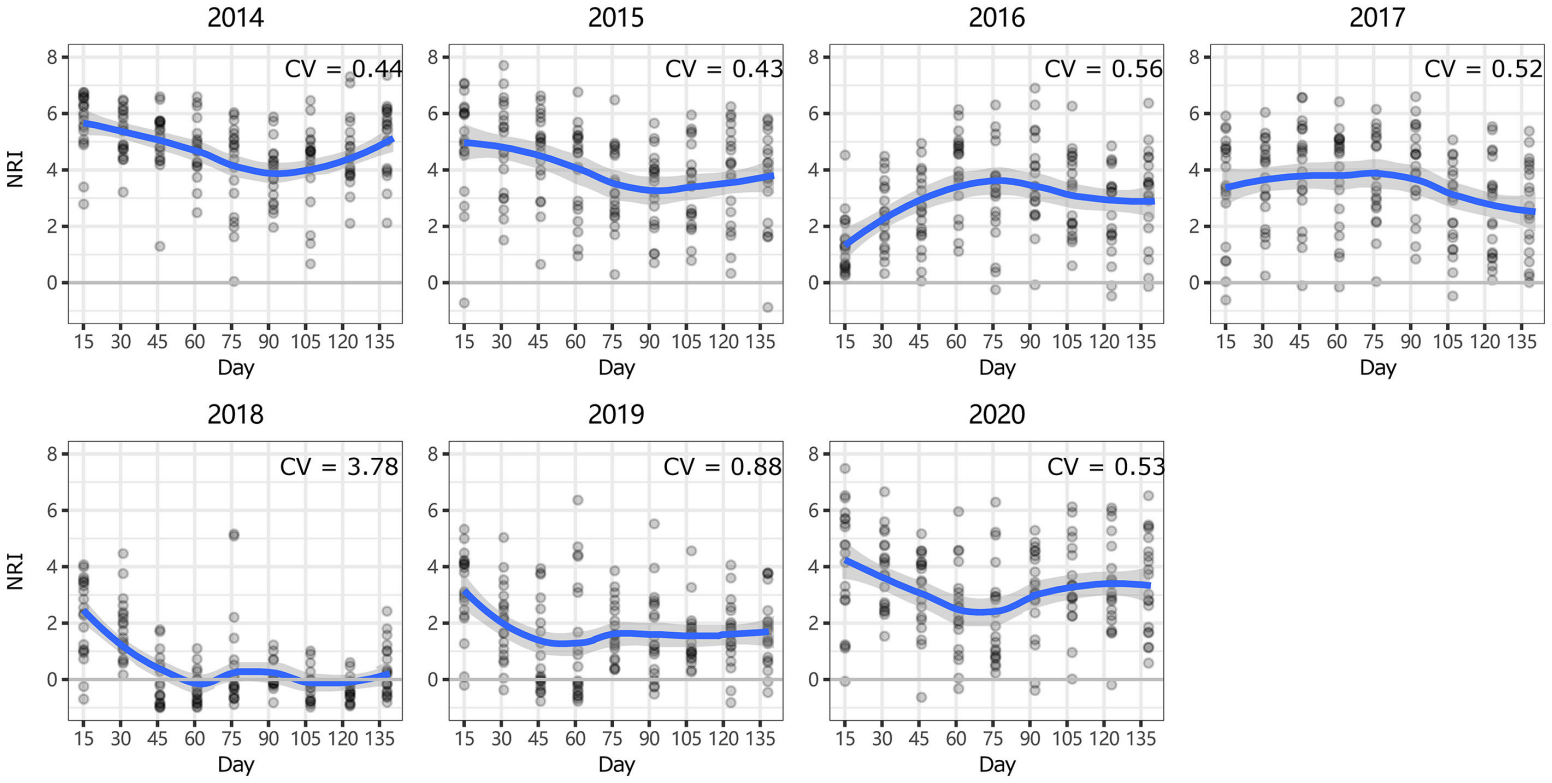
Intra-annual dynamics of community phylogenetic structure at the Inner Mongolia Grassland Ecosystem Research Station (ERS) in Inner Mongolia, China. The ordinate shows the net relatedness index (NRI). The abscissa shows the number of days since May 1. The blue line indicates the model prediction. The gray area shows the 95% confidence interval around the mean.

**Figure 5:**
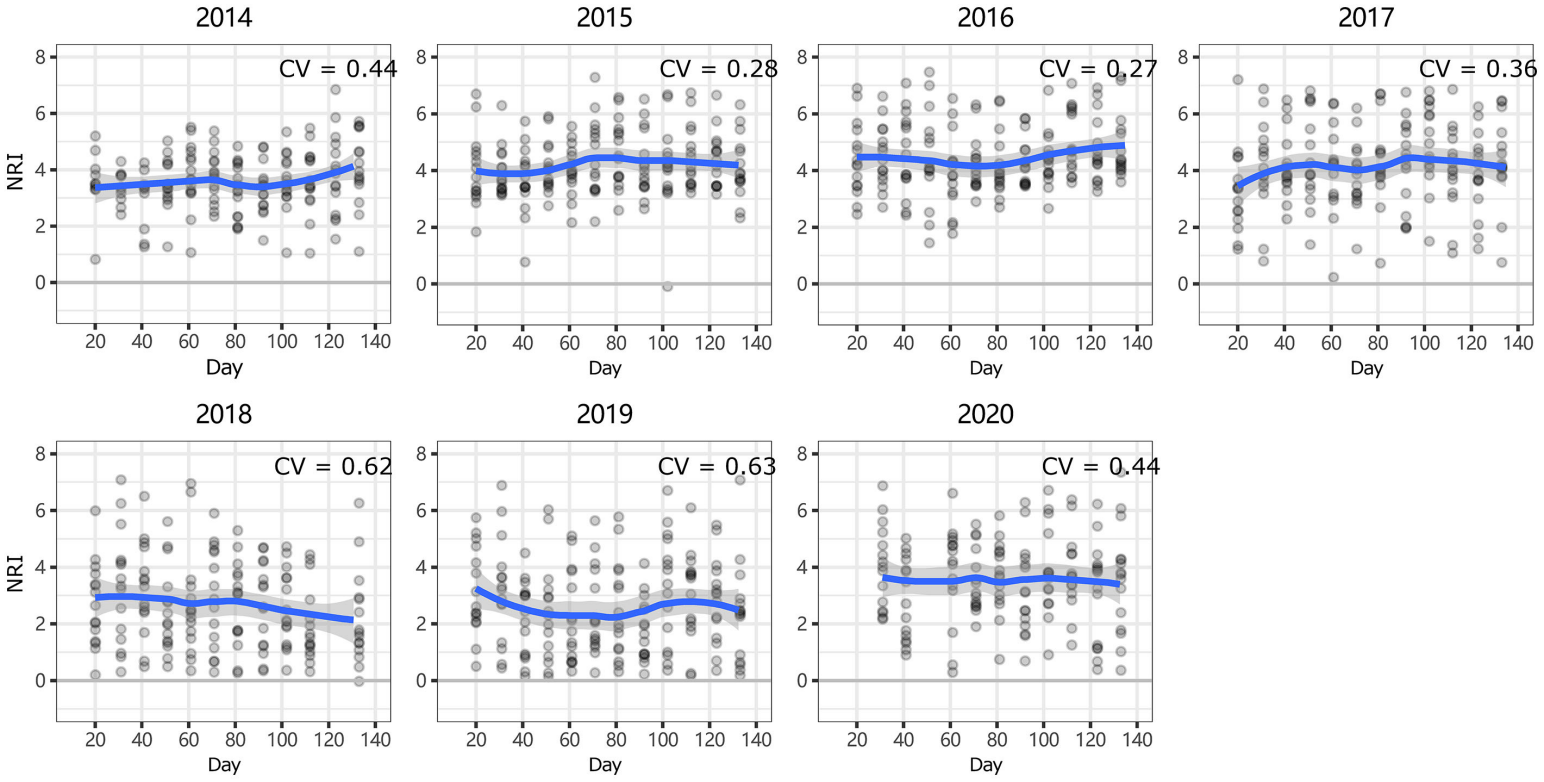
Intra-annual dynamics of community phylogenetic structure at the Xilinhot National Climate Observatory (NCO) in Inner Mongolia, China. The ordinate shows the net relatedness index (NRI). The abscissa shows the number of days since May 1. The blue line indicates the model prediction. The gray area shows the 95% confidence interval around the mean.

At ERS, the intra-annual NRI exhibited an inverse unimodal curve (except in 2016 and 2017); that is, the NRIs were the highest at the beginning of the growing season and tended to decrease as the season progressed but then increased again at the end of the growing season (only significant in 2014 and 2018, **Figure S3**). In 2016, the NRIs tended to increase from the beginning of the growing season until the middle of the growing season and became inversely unimodal (**Figure 4**). At NCO, the NRIs did not show a clear intra-annual trend. Only in 2014, 2016, and 2017, the NRIs were higher in the late-middle stages of the growing season than in the early-middle stages of the growing season (**Figure 5** and **Figure S4**).

### Interannual dynamics

**Figure 6:**
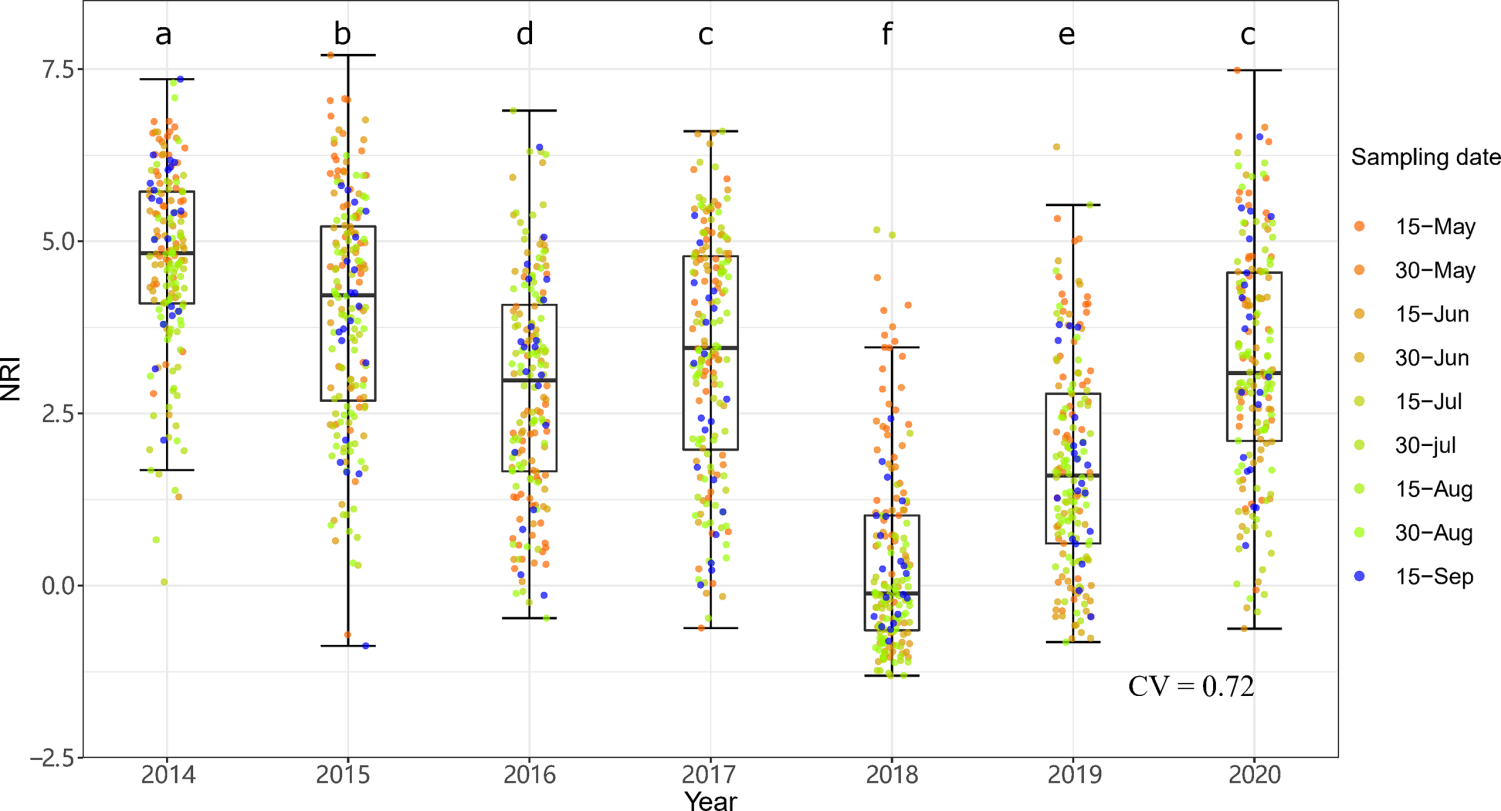
Interannual dynamics of community phylogenetic structure at the Inner Mongolia Grassland Ecosystem Research Station (ERS) in Inner Mongolia, China. The differences in the annual NRIs are denoted by different letters according to Duncan’s *post hoc* test (*p* < 0.05).

**Figure 7:**
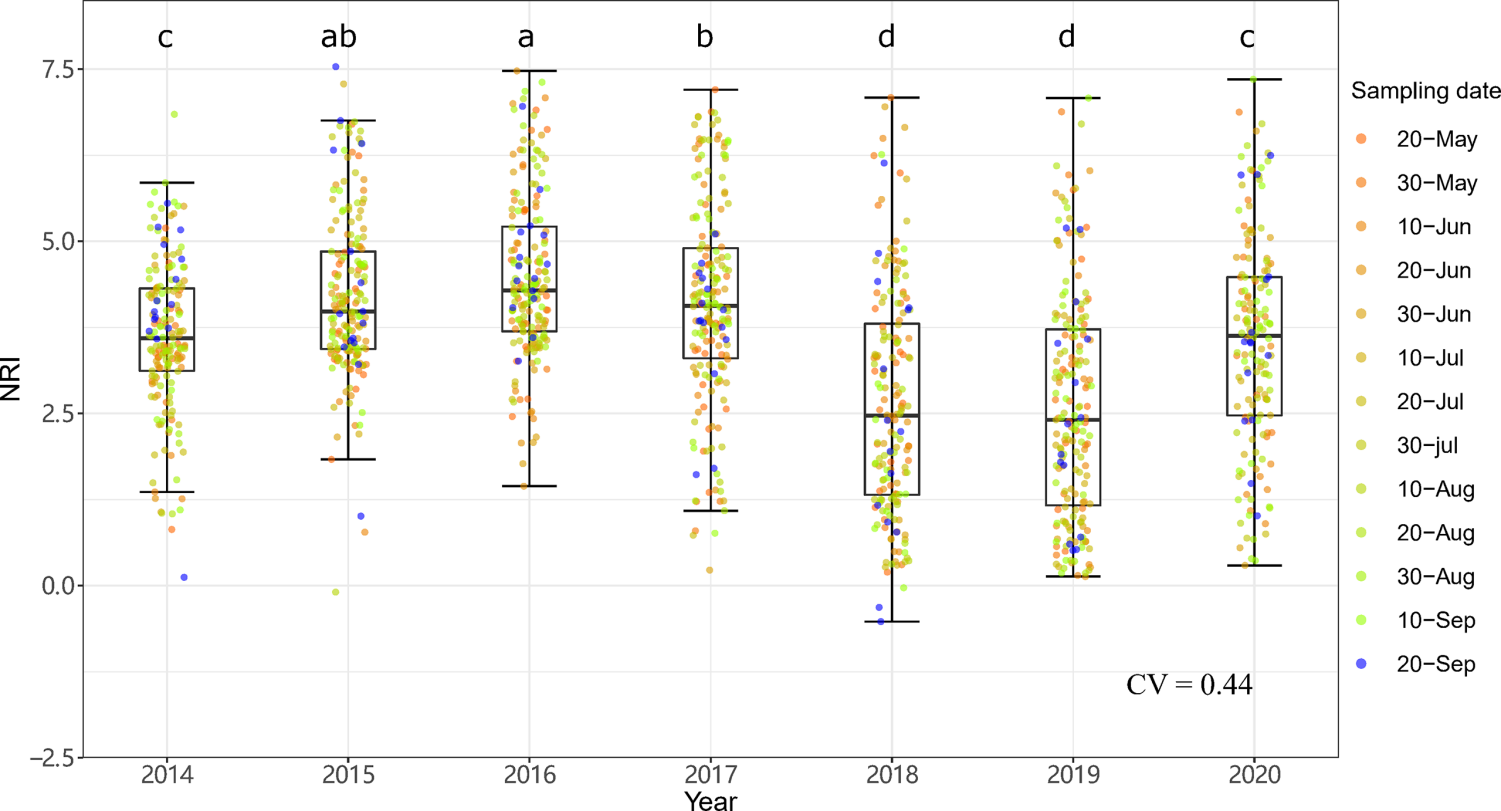
Interannual dynamics of community phylogenetic structure at the Xilinhot National Climate Observatory (NCO) in Inner Mongolia, China. The differences in the annual NRIs are denoted by different letters according to Duncan’s *post hoc* test (*p* < 0.05).

The community phylogenetic structure at both sites showed significant interannual variation based on one-way ANOVA. At ERS, the annual mean NRIs showed an inverse unimodal curve from 2014 to 2020. The annual mean NRIs in 2018 (i.e., the year following the drought year) were the lowest, followed by those in 2019 (**Figure 6**). The annual mean NRIs were the highest in 2014. At NCO, the lowest annual mean NRI values were also observed in 2018 and 2019, and the highest annual mean NRI values were observed in 2015 and then in 2016 (**Figure 7**).

### Effects of meteorological conditions on community phylogenetic structure

Multiple regression showed that meteorological factors before the current growing season accounted for 71.12% of the variation in the NRI at ERS. Among these factors, the average temperature in January (*T*_Jan_) and lower precipitation in the last 2 years (*P*_less2_) accounted for 15.53% and 52.20% of the variation, respectively. In addition, the interaction between *T*_min_ and *P*_less2_ accounted for 1.98% of the variation in the NRI. Meanwhile, the meteorological factors of the current growing season (i.e., CAP, CAT0, and *T*_15_) accounted for only 7.48% of the variation in the NRI (**Table 1**).

**Table 1:** Multiple regression analyses of the relationships between community phylogenetic structure and meteorological data at Inner Mongolia Grassland Ecosystem Research Station (ERS) in Inner Mongolia, China.

Factors	*Df*	MS	SS (%)	*F* value
*T*_Jan_	1	9.63	15.53**	39.92
*P*_less2_	1	32.37	52.20**	134.19
*T*_min_	1	0.88	1.41	3.63
CAP	1	0.79	1.27	3.27
CAT0	1	3.31	5.35**	13.74
*T*_15_	1	0.54	0.86	2.22
*T*_min_:*P*_less2_	1	1.23	1.98*	5.08
Residuals	55	0.24	21.39	–

Precipitation of the past years (P_past_) was removed before fitting the regression model because of high multicollinearity (variance inflation factor > 10).

MS, mean squares; SS, proportion of variance explained by the variable; T_Jan_, average temperature in January; P_less2_, precipitation of the years with less precipitation in the past 2 years; T_min_, extremely low temperature in January; CAP, current accumulated precipitation from January 1 until the sampling day; CAT0, current accumulated temperature >0°C from January 1 until the sampling day; T_15_, average temperature of the last 15 days before sampling; T_min_:P_less2_, interaction between T_min_ and P_lesst2_.

*p < 0.05; **p < 0.01.

Multiple regression showed similar results at NCO. Meteorological factors before the current growing season (i.e., *T*_Jan_ and *P*_less2_) accounted for 61.20% of the variation in the NRI. Current accumulated precipitation (CAP) and current accumulated temperature >0°C (CAT0) accounted for 1.23% and 1.09% of the variation in the NRI, respectively (**Table 2**).

**Table 2:** Multiple regression analyses of the relationships between community phylogenetic structure and meteorological data at Xilinhot National Climate Observatory (NCO) in Inner Mongolia, China.

Factors	*Df*	MS	SS (%)	*F*
*T*_Jan_	1	6.27	7.21**	16.40
*P*_less2_	1	46.97	53.99**	122.81
CAP	1	1.07	1.23	2.79
CAT0	1	0.95	1.09	2.47
Residuals	57	0.38	36.49	–

MS, mean squares; SS, proportion of variances explained by the variable; T_Jan_, the average temperature in January; P_less2_, precipitation of the years with less precipitation in the past 2 years; CAP, current accumulated precipitation from January 1 until the sampling day; CAT0, current accumulated temperature >0°C from January 1 until the sampling day.

**p < 0.01.

Our SEMs fit the meteorological data well (*χ* ^2^ = 2.987, *p* = 0.394, GFI = 0.985, SRMR = 0.020, RMSEA < 0.001 and *χ* ^2^ = 0.852, *p* = 0.837, GFI = 0.997, SRMR = 0.017, RMSEA < 0.001 at ERS and NCO, respectively). In general, meteorological factors had only a slight direct impact on the phylogenetic structure at both sites. However, meteorological factors showed a strong indirect effect on the NRI *via* the proportion of perennial grass biomass (P.Grass).

**Figure 8:**
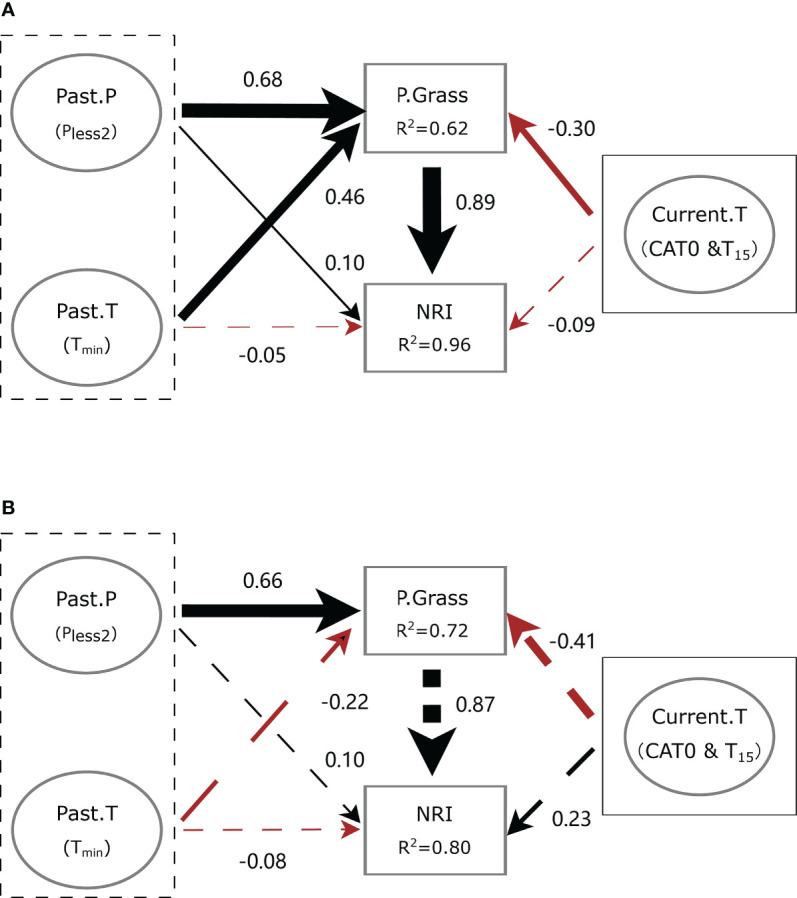
Simplified diagram of the structural equation model (SEM) showing the interplay between meteorological conditions and community phylogenetic structure at **(A)** the Inner Mongolia Grassland Ecosystem Research Station (ERS) and **(B)** Xilinhot National Climate Observatory (NCO) in Inner Mongolia, China. Black dotted boxes denote meteorological conditions before the growing season, and solid boxes denote meteorological conditions of the current growing season. Past.P indicates a precipitation factor before the growing season (i.e., precipitation of the years with less precipitation in the past 2 years, *P*_less2_). Past.T indicates a temperature factor before the growing season (i.e., extremely low temperature in January, *T*_min_). Current.T indicates meteorological factors of the current growing season, including accumulated temperature >0°C (CAT0) and average temperature of the last 15 days before sampling (*T*_15_). P.Grass, proportion of perennial grass biomass in a quadrat. NRI, net relatedness index. Numbers near the pathway arrows are the standard path coefficients. Black and red arrows represent positive and negative pathways, respectively. Dashed arrows indicate non-significant relationships (*p* > 0.05). Arrow width represents the strength of the relationship.

At ERS, *P*_less2_ and *T*_min_ had positive and negative direct effects on the NRI value, respectively (standardized path coefficient, *β* = 0.10, *p* = 0.03 and *β* = *−*0.05, *p* = 0.24, respectively). Notably, *P*_less2_ and *T*_min_ had significant positive direct effects on the proportion of perennial grass biomass in the community (i.e., P.Grass, *β* = 0.68 and 0.46, respectively). Meteorological conditions of the current growing season (Current.T, including CAT0 and *T*_15_) had non-significant direct negative effects on the NRI value (*β* = *−*0.09, *p* = 0.09). In addition, the indirect effects of *P*_lesst2_, *T*_Jan_, and Current.T *via* P.Grass on the NRI were 0.60 (*p* < 0.01), 0.40 (*p* < 0.01), and −0.26 (*p* = 0.02), respectively (**Figure 8A**).

At NCO, *P*_less2_ and *T*_min_ had non-significant positive and negative direct effects on the NRI value (*β* = 0.10, *p* = 0.74 and *β* = *−*0.08, *p* = 0.74, respectively). Meanwhile, Current.T had a non-significant direct effect on the NRI value (*β* = 0.23, *p* = 0.72). The indirect effect of *P*_less2_ on the NRI was 0.57 (*p* = 0.04). However, the indirect effects of *T*_min_ and Current.T on the NRI were not significant (*β* = −0.19, *p* = 0.55 and *β* = 0.36, *p* = 0.67, respectively) (**Figure 8B**).

### Long-term effects of meteorological conditions on community phylogenetic structure

**Figure 9:**
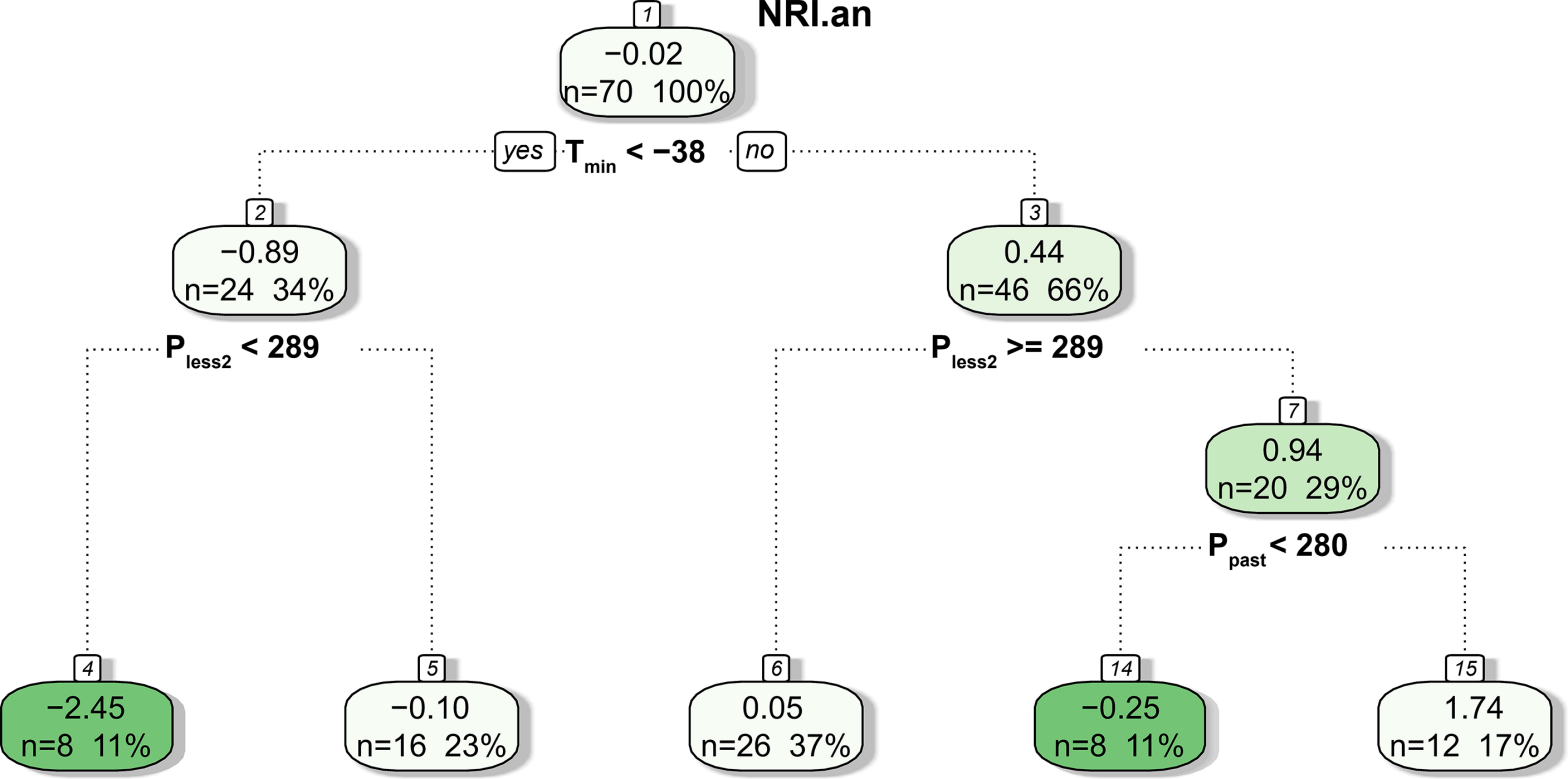
Result of the CART analysis. The values in each box are the mean NRI_an_, the number of predicted outcomes, and the percentage of predictions relative to the total number of samples.

The CART results showed that only meteorological factors before the growing season (i.e., *T*_min_, *P*_less2_, and *P*_past_) were used in tree construction, and NRI_an_ was mainly related to *T*_min_ and *P*_less2_ (**Figure 9**). Specifically, NRI_an_ was divided into two branches by a *T*_min_ of −38°C; that is, *T*_min_ <−38°C resulted in a negative NRI_an_ (node 2, mean NRI_an_ = −0.89), and *T*_min_ ≥−38°C resulted in a positive NRI_an_ (node 3, mean NRI_an_ = 0.44). In addition, the tree was further divided into five branches according to *P*_less2_ and *P*_past_. Notably, the interaction between *T*_min_ <−38°C and *P*_less2_ <289 mm resulted in the lowest NRI_an_ (mean = −2.45, *n* = 8), followed by the interaction between *T*_min_ ≥−38°C and *P*_past_ *<*280 mm (mean = −0.25, *n* = 8).

The LMM results showed that only *T*_min_ and the random factor (i.e., year) were significantly correlated with NRI_an_ (*p* = 0.01, **Figure S6C**). LMMs also showed that the amount of variation in NRI_an_ explained by the random factor (i.e., year) was greater than that explained by fixed factors (e.g., *T*_min_) (**Table 3**).

**Table 3:** Linear mixed-effects models (LMMs) between NRI_an_ and meteorological factors.

Model	Factor type	Factor	Mean Sq	*F* value	*p*	Marginal *R* ^2^	Conditional *R* ^2^
Meteorological factors before the growing season	Fixed factors	*T*_min_	3.94	7.04	0.01	0.20	0.77
		*T*_Jan_	0.63	1.12	0.30		
		*P*_less2_	<0.01	0.01	0.94		
		*P*_past_	0.85	1.52	0.23		
	Random factor	Year	–	–	<0.01	–	
Meteorological factors of the current growing season	Fixed factors	ACT0	<0.01	1.00	0.32	0.03	0.82
		ACP	<0.01	0.29	0.59		
		*T*_15_	<0.01	0.03	0.87		
		P_15_	<0.01	3.99	0.05		
	Random factor	Year	–	–	<0.01	–	

Marginal R^2^, variance explained by the fixed effects; conditional R^2^, variance explained by the entire model.

## Discussion

### Community phylogenetic structure and intra- and interannual dynamics

At both sites, the NRI was significantly greater than 0, indicating phylogenetic clustering (Webb et al., 2002). There are two processes that may result in phylogenetic clustering (under the assumption of niche conservatism) (Webb et al., 2002; Donoghue, 2008; Mayfield and Levine, 2010), habitat filtering (i.e., abiotic filtering), and competitive exclusion between distantly related phylogenetic clades because of competitive ability differences (i.e., biotic filtering) (Mayfield and Levine, 2010; Goberna et al., 2015), although it is difficult to detect abiotic and biotic filtering by phylogenetic analyses alone (Swenson and Enquist, 2009). Our results showed that the NRI was highly positively correlated with the proportion of perennial grass (**Figures S5**, **S6A**). This suggests that the relative competitive advantage of grasses over forbs and shrubs is an important driver of community phylogenetic clustering. Meanwhile, the community phylogenetic structures of both sites were related to meteorological factors, especially considering that the degree of clustering of community phylogenetic structure in early spring and autumn was higher (**Figures 4**, **5**; see below), suggesting that abiotic filtering factors may also have an impact on community phylogenetic structure. However, given that life strategies and competitive abilities are strictly related to the environment, abiotic filtering factors may still be the major driver of community phylogenetic clustering (Saito et al., 2016). Our results were partly consistent with those of Yang et al. (2018), who found that in an undisturbed (i.e., control treatment) Inner Mongolian grassland, the community showed a positive NRI (although not significantly different from 0), and nitrogen addition promoted the colonization of species distantly related to the residents. Qian et al. (2019) also found that, in general, the angiosperm community showed phylogenetic clustering in northwestern China.

In general, the intra-annual NRI showed an inverse unimodal curve at ERS (**Figure 4**). This is consistent with our hypothesis 1 that habitat filtering would play a greater role in community assembly at the beginning of the growing season than in the peak growing season. The reason may be related to the harsh environment at the beginning and end of the growing season. To avoid facing low temperature and precipitation, some species (e.g., *Heteropappus altaicus*) delay revival at the beginning of the growing season. For the same reason, some species (especially annual plants, e.g., *Dontostemon dentatus*) complete their life cycle before the autumn arrives. That is, competition between species is most intense during the peak growing season (i.e., July to August) (Doležal et al., 2019) when the environmental filtering effect is the smallest. As a result, the community showed less phylogenetic clustering during the peak growing season.

Notably, both the intra- and interannual variability (indicated by coefficients of variation, CVs) at ERS were greater than those at NCO (**Figures 4** **–** **7**). This result indicates that the community phylogenetic structure at NCO is more stable than that at ERS, which has more species richness than the former (**Figure 3B**). The relationship between biodiversity and ecosystem stability remains a controversial topic (Fonseca and Ganade, 2001; Mazzochini et al., 2019). Traditionally, more ecologists believe that biodiversity is positively related to ecosystem stability (i.e., resistance and resilience) because larger species pools can contain more species and functional groups that adapt to different disturbances (McNaughton, 1978; Yachi and Loreau, 1999). In recent years, increasing evidence has shown that biodiversity may also be negatively correlated with ecosystem stability. For instance, Pfisterer and Schmid (2002) found that species-poor systems were more resistant to disturbance than species-rich systems. Van Ruijven and Berendse (2010) found that biodiversity enhances grassland community recovery, but its relationship with resistance is highly dependent on biomass. However, previous studies mainly used the dynamics of biomass as an indicator of community resistance and resilience to perturbation (Tilman and Downing, 1994; Tilman et al., 2006; Isbell et al., 2015). Here, we provide a new perspective on how biodiversity affects ecosystem stability. That is, in view of community phylogenetic structure, we provide evidence that communities with low biodiversity may have higher stability than communities with high biodiversity.

### Impact of climate factors on community phylogenetic structure

Our results showed that meteorological factors before the growing season explained more of the variation in the NRI and NRI_an_ than meteorological factors during the current growing season on both short-term and long-term scales (**Tables 1** **–** **3**, **Figures 8**, **9**). Vegetation and community production show a time-lag response to climate that has been widely reported (Bradley et al., 1999; Piao et al., 2003; Bai et al., 2004; Vicente-Serrano et al., 2013; Wu et al., 2015); however, the lag response of community phylogenetic structure still receives little attention. Here, we provide evidence that the dynamics of grassland community phylogenetic structure are deeply influenced by meteorological conditions before the growing season rather than during the current growing season, which suggests the time lag of the community phylogenetic structure responses to meteorological factors (i.e., hypothesis 2).

Our results highlight the fact that extreme climatic events, such as drought and extreme cold, may impact grassland community structure and reduce the degree of phylogenetic clustering. Additionally, the SEMs showed that meteorological factors mainly indirectly changed community phylogenetic structure by influencing the proportion of perennial grass biomass (**Figure 8**). As we mentioned above, the competitive advantage of grasses is one of the key reasons why the community remains phylogenetically clustered. In other words, drought and extreme cold inhibit the dominance of grass (**Figure 8**). We found that after drought years (i.e., in 2018 and 2019), the lower NRIs at ERS were mainly related to an abnormal decrease in perennial grasses and an increase in forbs (e.g., *Potentilla bifurca* and *Klasea centauroides*) in the community. In contrast, due to the lack of perennial forbs, niches were occupied by annual forbs (e.g., *Salsola collina* and *Dysphania aristata*) at NCO. This was consistent with the finding of VanderWeide and Hartnett (2015) that drought decreased grass but not sedge and forb bud and shoot densities. Bai et al. (2004) found a negative correlation between dominant grass species and non-dominant species in a typical steppe, which is a key mechanism maintaining the stability of the ecosystem. Drought or extreme cold may decrease the proportion of perennial grass because grasses and forbs have different reproduction strategies. Specifically, compared to grasses, forbs rely more on sexual reproduction (Song et al., 2001; Stampfli and Zeiter, 2004), and seeds are more likely to survive low temperatures and in other unfavorable environments than buds (Carter et al., 2012). Stampfli and Zeiter (2004) suggested that recolonization from seeds was the key to recovery after interference. However, the sexual reproduction ability of dominant grasses in the Mongolian Plateau (i.e., *S. grandis* and *L. chinensis*) is usually lacking (Teng et al., 2006). Therefore, extreme weather events are more likely to lead to the death of perennial grasses. In addition, plants tend to allocate more resources to sexual reproduction under resource shortages or in unfavorable habitats (Olejniczak, 2001; Dong et al., 2015). As a result, extreme meteorological events before the growing season inhibited the reproduction of grasses and further reduced the degree of phylogenetic clustering.

Our results were consistent with those of Evans et al. (2011), who found that drought may result in a reduction in dominant grass and correspond with an increase in forbs. The authors attributed this to the higher colonization ability of forbs under drought conditions (Evans et al., 2011). Other researchers have suggested that grass generally has shallow and fibrous roots and thus is more sensitive to drought than forbs with deep roots and taproots (Buckland et al., 1997; Dunnett et al., 1998). In addition, in Mediterranean grasslands, researchers found that both dominant perennial grasses and annuals increased substantially after extreme events (such as burning), but the latter increased even faster for the reason of surviving as seeds (Vidaller et al., 2019). As a result, when faced with an unfavorable environment, perennial grasses in grasslands may be more affected, while forbs and annuals occupy the niche vacancies of perennial grasses, thus changing community species composition and affecting community phylogenetic structure.

Notably, at both sites, the NRIs in 2018 and 2019 were significantly lower than those in other years (**Figures 6**, **7**). Multiple regression showed that precipitation of the years with less precipitation in the past 2 years (i.e., *P*_less2_) explained more variation than precipitation of the last year (**Tables S1**, **S2**). This implies that the effects of drought last more than 1 year. Our results were consistent with those of VanderWeide et al. (2014), who found that the impact of drought on grasslands may last more than 1 year. Oesterheld et al. (2001) also found that interannual variation in primary production of a semiarid grassland was related to previous-year production. The authors suggested that seed, plant, and tiller density might be reduced after a drought year with low precipitation and, thus, might either constrain the ability of ecosystems to respond to a subsequent increase in water availability (Oesterheld et al., 2001).

We found that extremely low temperature (i.e., *T*_Jan_ and *T*_min_) contributed to the reduction in phylogenetic clustering, especially on a long time scale (**Figure 9** and **Table 3**). However, extremely low temperature had a significant influence on NRI_an_ rather than the NRI, suggesting that community phylogenetic structure depended on not only meteorological conditions but also the community structure of the last year. LMM analyses additionally showed that the year explained more of the variation in NRI_an_ than meteorological factors (the random factor explained 0.77 and 0.82 of the variation in NRI_an_ at ERS and NCO, respectively). That is, the interannual dynamics of community phylogenetic structure are obvious regardless of changes in climatic conditions. Therefore, grassland community phylogenetic structure should not be treated as quiescent, and assessment of specific grassland communities’ phylogenetic structure needs to include a multiyear average rather than one sampling; in particular, the impact of extreme meteorological events before sampling needs to be fully considered.

Our results were partially consistent with those of Zheng et al. (2019), who found that winter temperature was a major factor leading to phylogenetic overdispersion of the shrub community on the Mongolian Plateau. Our results suggested that extremely low temperatures play an important role in steppe biodiversity by inhibiting the competitive ability of grass. Therefore, with global climate warming, the reduction in extreme low-temperature events (i.e., frost days; see Sillmann et al., 2013) may adversely affect grassland biodiversity. The impact of extreme climate conditions on grassland community structure still needs more exploration.

## Conclusions

In this study, we provide evidence that grassland community phylogenetic structure varies intra- and interannually and that its dynamics are influenced by meteorological conditions before the growing season. The degree of phylogenetic clustering of the community is relatively low in the peak growing season, when habitat filtering is relatively weak and competition is fiercer. In addition, extreme climatic events, such as drought and extreme cold, may indirectly reduce the degree of phylogenetic clustering by reducing the proportion of perennial grass. In the future, global climate change may alter grassland community phylogenetic structure and assembly patterns and further reduce community biodiversity.

## Data availability statement

The original contributions presented in the study are included in the article/**Supplementary Material**. Further inquiries can be directed to the corresponding author.

## Author contributions

LD and CL collected the data. LD and YZ analyzed the data. LD and YZ drafted the manuscript. All authors contributed to the article and approved the submitted version.

## Funding

This study was supported by the Planned Science-Technology Project of Inner Mongolia, China (2021GG0050); Nature Science Foundation of Inner Mongolia (2022QN03010), the Special Project of Basic Scientific Research Business Expenses of China Institute of Water Resources and Hydropower Research (MK2021J11); and the National Key Research and Development Program of China (2016YFC0500503).

## Acknowledgments

The authors are indebted to the members of the Inner Mongolia Grassland Ecosystem Research Station (IMGERS) and Xilinhot National Climate Observatory (XNCO) who helped collect the samples.

## Conflict of interest

The authors declare that the research was conducted in the absence of any commercial or financial relationships that could be construed as a potential conflict of interest.

## Publisher’s note

All claims expressed in this article are solely those of the authors and do not necessarily represent those of their affiliated organizations, or those of the publisher, the editors and the reviewers. Any product that may be evaluated in this article, or claim that may be made by its manufacturer, is not guaranteed or endorsed by the publisher.
