# Supplementary material for: Intra- and interannual dynamics of grassland community phylogenetic structure are influenced by meteorological conditions before the growing season
Source: Front Plant Sci. 2022 Sep 23;13:870526. doi: 10.3389/fpls.2022.870526 (PMC9541524; doi:10.3389/fpls.2022.870526)
Supplement: Supplementary file 1 [file DataSheet_1.pdf]

SUPPORTING INFORTATION

SUPPLEMENTARY FIGURES

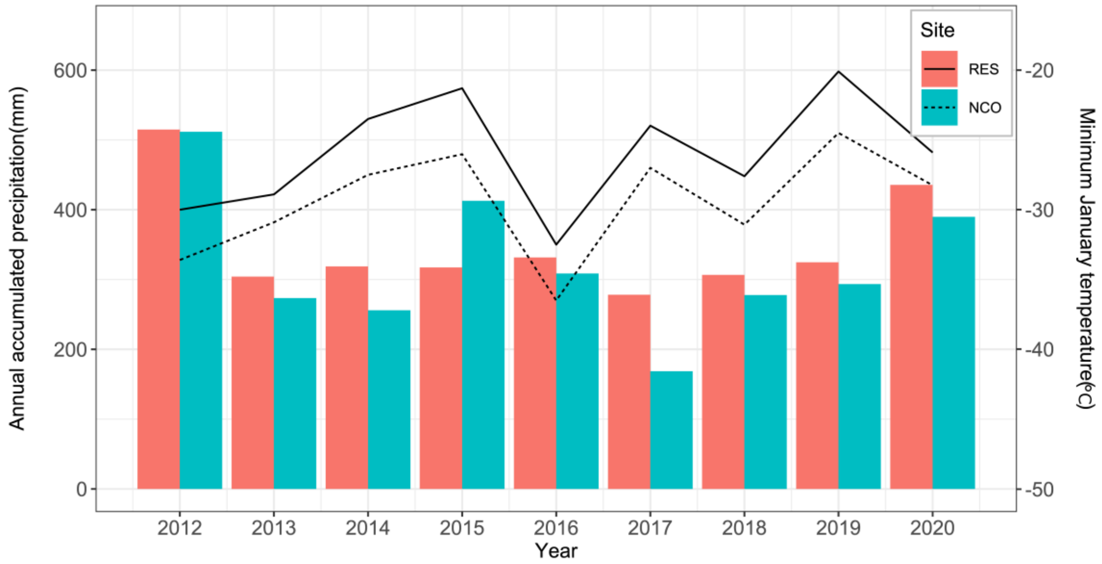

Fig. S1 Precipitation (bars) and minimum January temperature (lines) from 2012 to 2020.

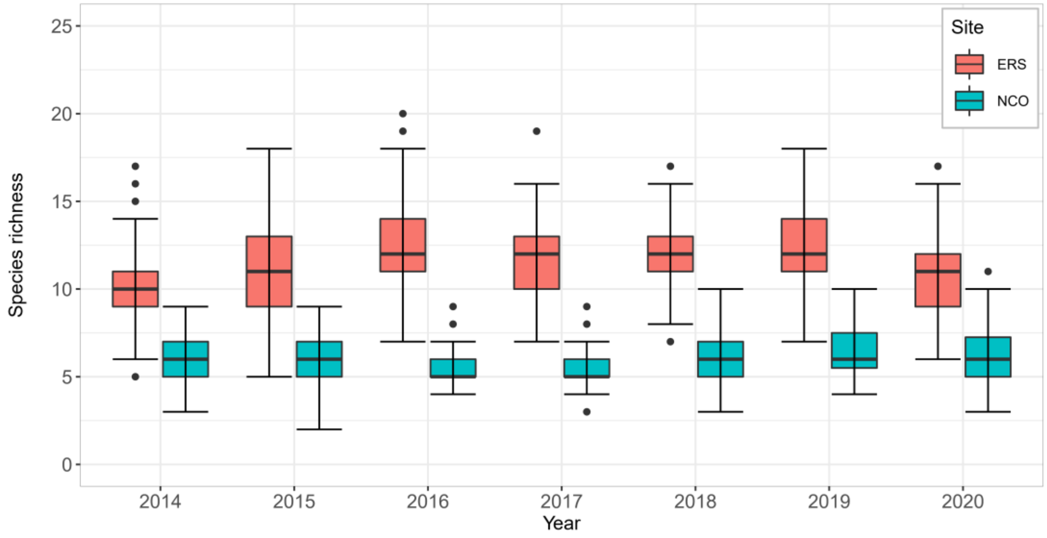

Fig. S2 Species richness at site ERS and NCO. Different upper-case and lower-case letters denotes significant differences at site ERS and NCO, respectively (Duncan's multiple range test, $P < 0.05$).

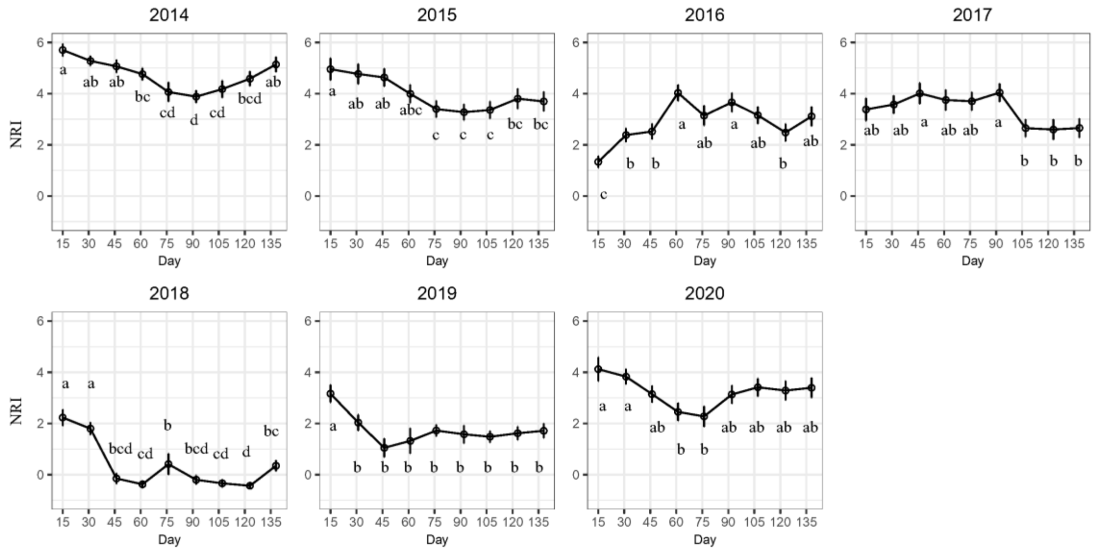

Figure S3 Intra-annual dynamic of community phylogenetic structure at site ERS. The abscissa indicates the number of days since May 1st and ordinate indicates net relatedness index (NRI). The differences ($P < 0.05$) among intra-annual NRIs are denotes by different letters according to Duncan's post hoc test.

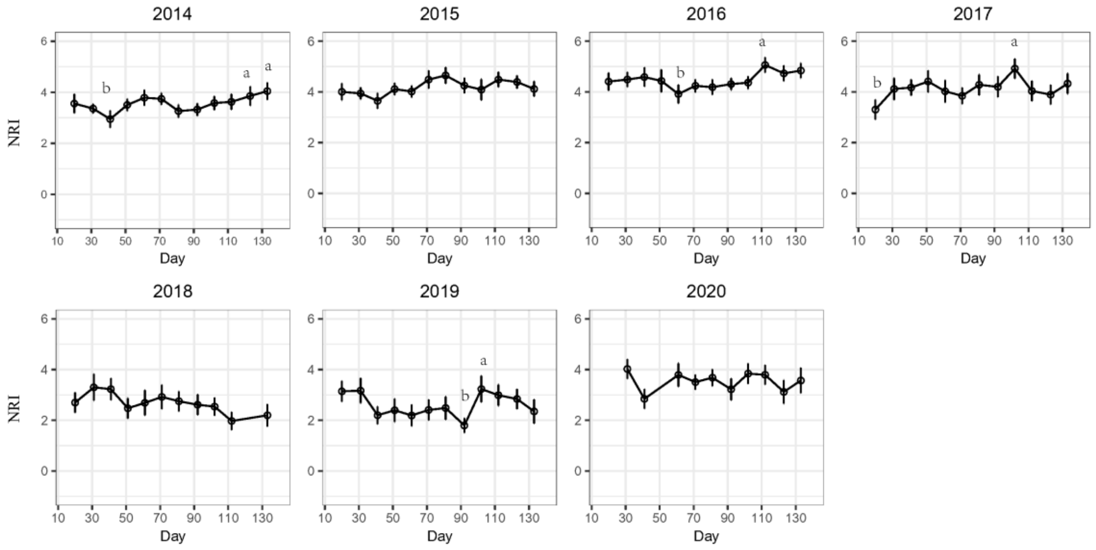

Figure S4 Intra-annual dynamic of community phylogenetic structure at site NCO. The abscissa indicates the number of days since May 1st and ordinate indicates net relatedness index (NRI). The differences ($P < 0.05$) among intra-annual NRIs are denotes by different letters according to Duncan's post hoc test.

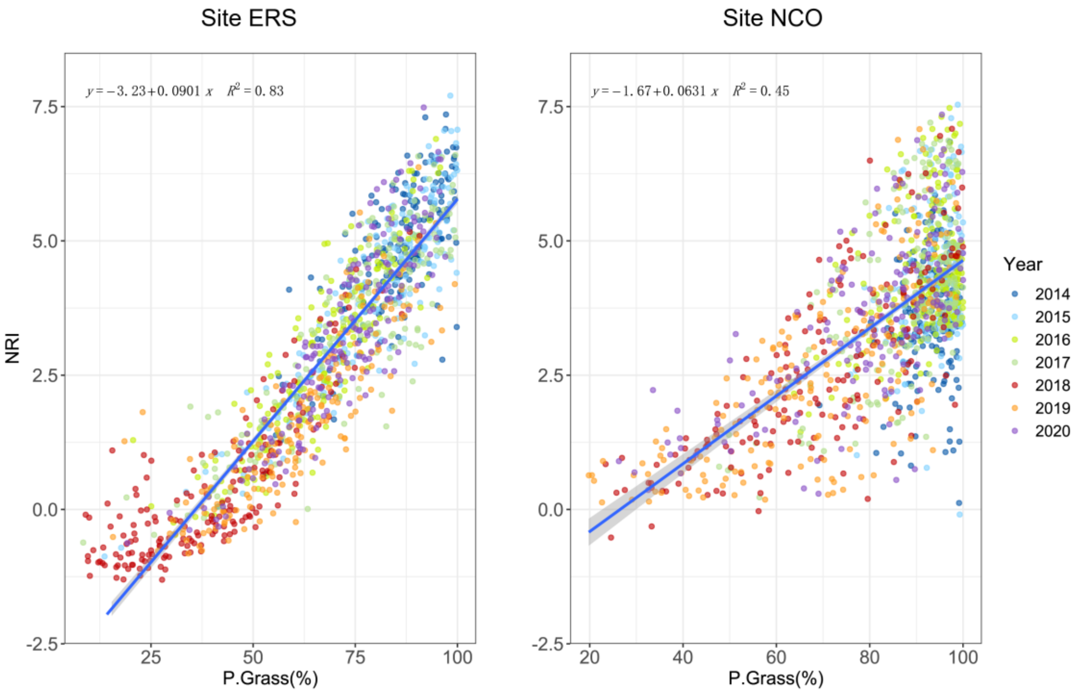

Fig. S5 Relationship between net relatedness index (NRI) and proportion of perennial grass (P.Grass). Blue line indicates the linear regressions. Gray area shows 95% confidence interval.

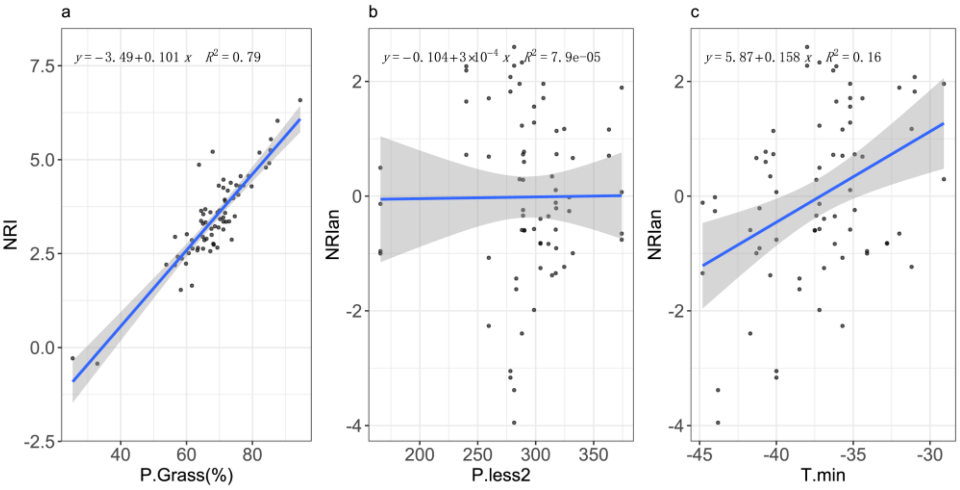

Fig. S6 Relationship between a) net relatedness index (NRI) and proportion of perennial grass (P.Grass), b) yearly net relatedness index anomaly (NRI_{an}) and precipitation of the years with less precipitation in the past two years ($P._{less2}$), c) yearly net relatedness index anomaly (NRI_{an}) and extremely low temperature in January ($T._{min}$) at ERS using long-term database. Blue line indicates the linear regressions. Gray area shows 95% confidence interval.

SUPPLEMENTARY TABLES

Table S1 Akaike information criterion (AIC) values of three best multiple regressions models of community phylogenetic structure against meteorological data at ERS. The best model (*i.e.*, the lowest AIC) is in bold.

Model	Factors	AIC	R^2	F	*P* value
A1	CAT0, T_{15}, T_{min} and P_{past}	-25.17	0.44	8.84	2.95e-06
A2	T15 and P_{past}	-28.85	0.42	21.33	1.005e-07
**B1**	**CAT0, T_{15}, T_{min} and P_{less2}**	**-51.94**	**0.62**	**23.64**	**< 1.25e-11**

Table S2 Akaike information criterion (AIC) values of three best multiple regressions models of community phylogenetic structure against meteorological data at NCO. The best model (*i.e.*, the lowest AIC) is in bold.

Model	Factors	AIC	R^2	F	*P* value
A1	CAP, CAT0, P_{past}	-41.42	0.45	13.35	1.607e-09
A2	CAP, CAT0, T_{min} and P_{past}	-43.03	0.45	16.73	4.322e-09
**B1**	**CAP, CAT0, T_{min} and P_{less2}**	**-74.25**	**0.62**	**26.80**	**5.827e-16**
