# Supplementary material for: Intra- and interannual dynamics of grassland community phylogenetic structure are influenced by meteorological conditions before the growing season
Source: Front Plant Sci. 2022 Sep 23;13:870526. doi: 10.3389/fpls.2022.870526 (PMC9541524; doi:10.3389/fpls.2022.870526)
Supplement: Supplementary file 2 [file DataSheet_2.docx]

# SUPPORTING INFORMATION 2

Intra-annual dynamic of community phylogenetic structure indicates nearest taxa index (NTI).

NTI was calculated as follows:

$$NTI=-1\times\frac{{MNPD}_{obs}-{MNPD}_{null}}{{sd(MNPD}_{null})}$$

where NTI is the nearest taxa index, MNPD_obs_ is the phylogenetic distance to the nearest taxon in the phylogeny (MNPD) in the quadrat, MNPD_null_ is 999 random MNPD values under the null model, and sd (MNPD_null_) is the standard deviation of MNPD_null_ (Webb *et al.* 2002; Swenson *et al.* 2007).


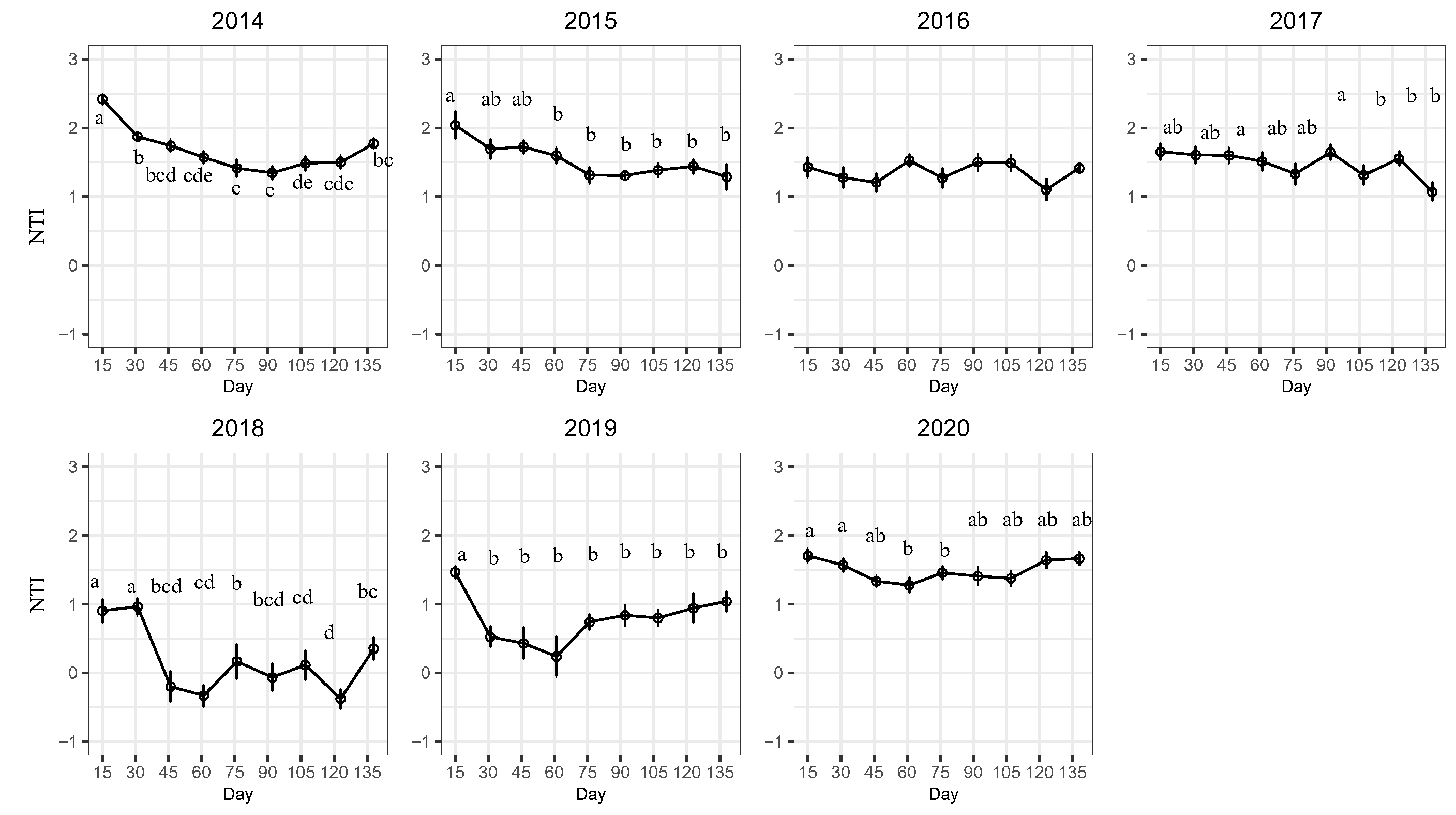


Figure S2-1 Intra-annual dynamic of community phylogenetic structure via. nearest taxa index (NTI) at site ERS. The abscissa indicates the number of days since May 1st and ordinate indicates nearest taxa index (NTI). The differences (*P* < 0.05) among intra-annual NRIs are denotes by different letters according to Duncan’s post hoc test.


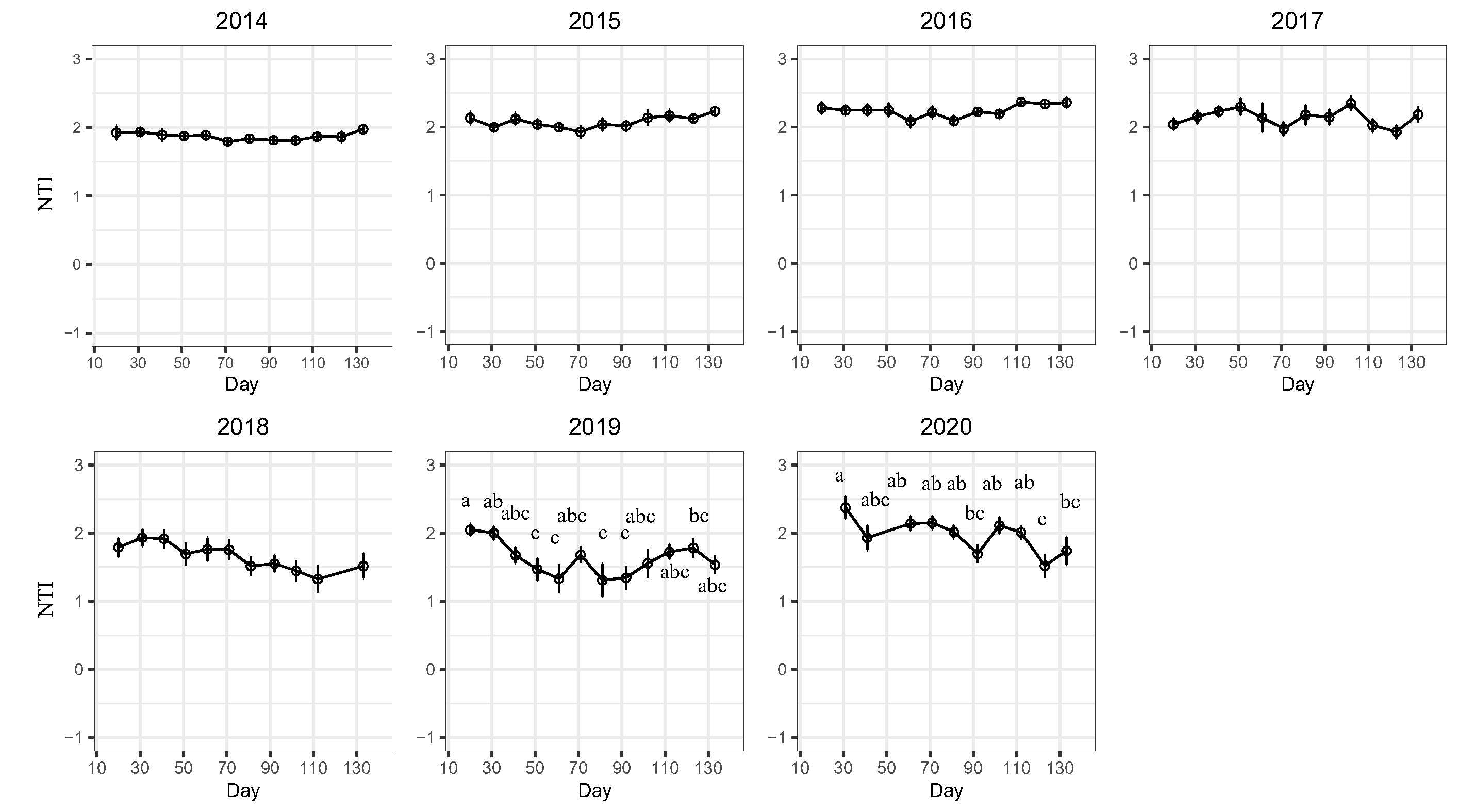


Figure S2-2 Intra-annual dynamic of community phylogenetic structure via. nearest taxa index (NTI) at site NCO. The abscissa indicates the number of days since May 1st and ordinate indicates nearest taxa index (NTI). The differences (*P* < 0.05) among intra-annual NRIs are denotes by different letters according to Duncan’s post hoc test.


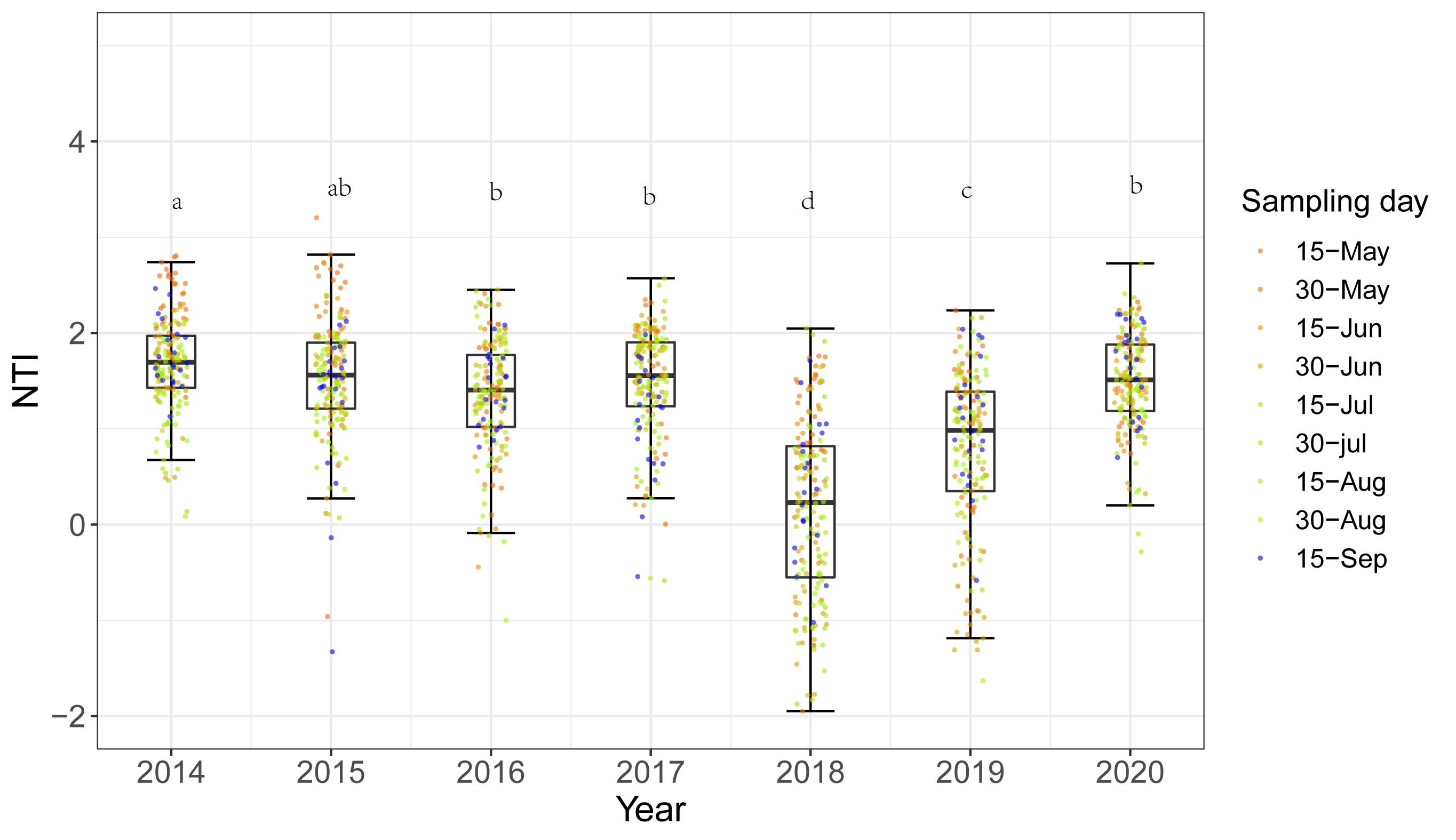


Figure S2-3 Inter-annual dynamics of community phylogenetic structure via. nearest taxa index (NTI) at the Inner Mongolia Grassland Ecosystem Research Station (ERS) in Inner Mongolia, China. The differences in the annual NTIs are denoted by different letters according to Duncan’s post hoc test (P < 0.05).


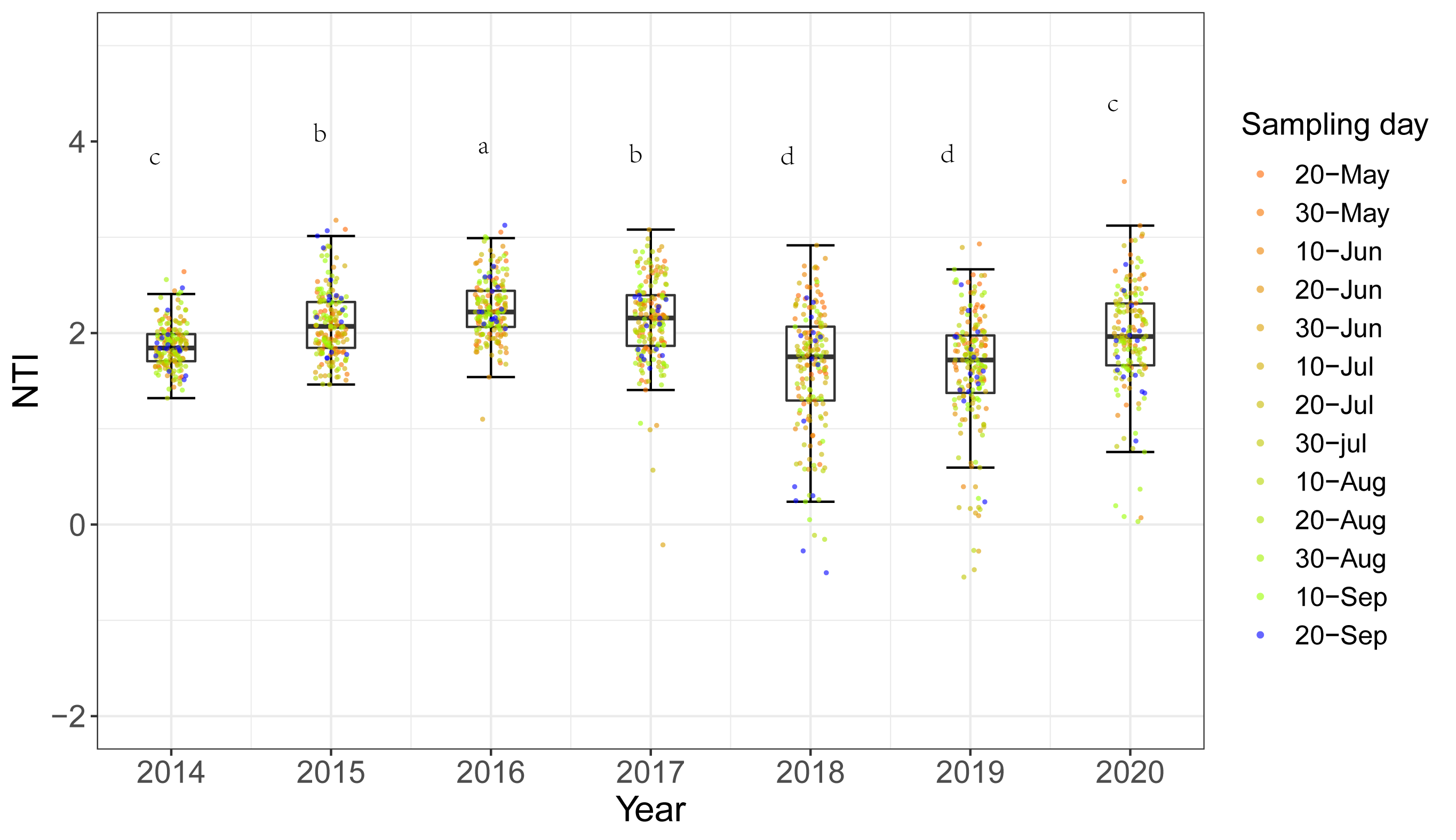


Figure S2-4 Inter-annual dynamics of community phylogenetic structure via. nearest taxa index (NTI) at the Xilinhot National Climate Observatory (NCO) in Inner Mongolia, China. The differences in the annual NTIs are denoted by different letters according to Duncan’s post hoc test (P < 0.05).

Table S2-1 List of all species included in this study according to APG Ⅳ (according to APG Ⅳ)

| Family | Genus | Species | ERS | NCO |
| --- | --- | --- | --- | --- |
| Amaranthaceae | *Axyris* | *Axyris amaranthoides* | * |  |
| Amaranthaceae | *Chenopodium* | *Chenopodium acuminatum* |  | * |
| Amaranthaceae | *Chenopodium* | *Chenopodium album* |  | * |
| Amaranthaceae | *Chenopodium* | *Chenopodium aristatum* | * |  |
| Amaranthaceae | *Chenopodium* | *Chenopodium glaucum* | * |  |
| Amaranthaceae | *Corispermum* | *Corispermum mongolicum* |  | * |
| Amaranthaceae | *Dysphania* | *Dysphania aristata* |  | * |
| Amaranthaceae | *Kochia* | *Kochia prostrata* | * |  |
| Amaranthaceae | *Salsola* | *Salsola collina* | * | * |
| Amaryllidaceae | *Allium* | *Allium anisopodium* | * | * |
| Amaryllidaceae | *Allium* | *Allium bidentatum* | * | * |
| Amaryllidaceae | *Allium* | *Allium condensatum* | * | * |
| Amaryllidaceae | *Allium* | *Allium mongolicum* |  | * |
| Amaryllidaceae | *Allium* | *Allium polyrhizum* |  | * |
| Amaryllidaceae | *Allium* | *Allium ramosum* | * | * |
| Amaryllidaceae | *Allium* | *Allium senescens* | * |  |
| Amaryllidaceae | *Allium* | *Allium tenuissimum* | * | * |
| Apiaceae | *Bupleurum* | *Bupleurum chinensis* | * |  |
| Apiaceae | *Saposhnikovia* | *Saposhnikovia divaricata* | * |  |
| Apocynaceae | *Cynanchum* | *Cynanchum thesioides* | * | * |
| Asparagaceae | *Anemarrhena* | *Anemarrhena asphodeloides* | * | * |
| Asteraceae | *Artemisia* | *Artemisia annua* | * | * |
| Asteraceae | *Artemisia* | *Artemisia argyi* | * |  |
| Asteraceae | *Artemisia* | *Artemisia frigida* | * |  |
| Asteraceae | *Artemisia* | *Artemisia pubescens* | * |  |
| Asteraceae | *Artemisia* | *Artemisia sieversiana* | * |  |
| Asteraceae | *Carduus* | *Carduus nutans* | * |  |
| Asteraceae | *Chrysanthemum* | *Chrysanthemum chanetii* | * |  |
| Asteraceae | *Erigeron* | *Erigeron acer* | * |  |
| Asteraceae | *Filifolium* | *Filifolium sibiricum* | * |  |
| Asteraceae | *Heteropappus* | *Heteropappus altaicus* | * |  |
| Asteraceae | *Ixeris* | *Ixeris polycephala* | * |  |
| Asteraceae | *Saussurea* | *Saussurea japonica* | * |  |
| Asteraceae | *Scorzonera* | *Scorzonera austriaca* |  | * |
| Asteraceae | *Serratula* | *Serratula centauroides* | * |  |
| Boraginaceae | *Lappula* | *Lappula myosotis* | * |  |
| Brassicaceae | *Dontostemon* | *Dontostemon dentatus* | * |  |
| Campanulaceae | *Adenophora* | *Adenophora stenanthina* | * |  |
| Caryophyllaceae | *Silene* | *Silene aprica* | * | * |
| Convolvulaceae | *Convolvulus* | *Convolvulus ammannii* |  | * |
| Convolvulaceae | *Convolvulus* | *Convolvulus arvensis* | * |  |
| Crassulaceae | *Orostachys* | *Orostachys malacophyllus* | * |  |
| Crassulaceae | *Sedum* | *Sedum aizoon* | * |  |
| Cyperaceae | *Carex* | *Carex korshinskyi* | * | * |
| Euphorbiaceae | *Euphorbia* | *Euphorbia esula* | * |  |
| Euphorbiaceae | *Euphorbia* | *Euphorbia humifusa* |  | * |
| Fabaceae | *Astragalus* | *Astragalus adsurgens* | * |  |
| Fabaceae | *Astragalus* | *Astragalus galactites* | * | * |
| Fabaceae | *Astragalus* | *Astragalus melilotoides* |  | * |
| Fabaceae | *Astragalus* | *Astragalus scaberrimus* |  | * |
| Fabaceae | *Caragana* | *Caragana microphylla* | * | * |
| Fabaceae | *Glycyrrhiza* | *Glycyrrhiza uralensis* | * |  |
| Fabaceae | *Gueldenstaedtia* | *Gueldenstaedtia verna* | * |  |
| Fabaceae | *Lespedeza* | *Lespedeza davurica* | * |  |
| Fabaceae | *Medicago* | *Medicago falcata* | * |  |
| Fabaceae | *Medicago* | *Medicago ruthenica* | * | * |
| Fabaceae | *Thermopsis* | *Thermopsis lanceolala* | * |  |
| Fabaceae | *Vicia* | *Vicia sepium* | * |  |
| Iridaceae | *Belamcanda* | *Belamcanda chinensis* | * |  |
| Iridaceae | *Iris* | *Iris tenuifolia* | * | * |
| Lamiaceae | *Phlomis* | *Phlomis umbrosa* | * | * |
| Lamiaceae | *Schizonepeta* | *Schizonepeta tenuifolia* | * |  |
| Lamiaceae | *Scutellaria* | *Scutellaria scordifolia* | * |  |
| Linaceae | *Linum* | *Linum stelleroides* | * |  |
| Orobanchaceae | *Cymbaria* | *Cymbaria daurica* | * | * |
| Orobanchaceae | *Pedicularis* | *Pedicularis striata* | * |  |
| Plantaginaceae | *Linaria* | *Linaria vulgaris* | * |  |
| Plumbaginaceae | *Limonium* | *Limonium bicolor* | * | * |
| Poaceae | *Achnatherum* | *Achnatherum sibiricum* | * | * |
| Poaceae | *Agropyron* | *Agropyron michnoi* | * |  |
| Poaceae | *Cleistogenes* | *Cleistogenes squarrosa* | * | * |
| Poaceae | *Koeleria* | *Koeleria litvinowii* | * |  |
| Poaceae | *Leymus* | *Leymus chinensis* | * | * |
| Poaceae | *Poa* | *Poa attenuata* | * |  |
| Poaceae | *Setaria* | *Setaria viridis* |  | * |
| Poaceae | *Stipa* | *Stipa grandis* | * | * |
| Polygonaceae | *Polygonum* | *Polygonum aviculare* | * |  |
| Portulacaceae | *Portulaca* | *Portulaca oleracea* |  | * |
| Ranunculaceae | *Pulsatilla* | *Pulsatilla chinensis* | * |  |
| Ranunculaceae | *Thalictrum* | *Thalictrum petaloideum* | * |  |
| Ranunculaceae | *Thalictrum* | *Thalictrum squarrosum* |  | * |
| Rosaceae | *Potentilla* | *Potentilla acaulis* | * |  |
| Rosaceae | *Potentilla* | *Potentilla bifurca* | * |  |
| Rosaceae | *Potentilla* | *Potentilla tanacetifolia* | * |  |
| Rosaceae | *Potentilla* | *Potentilla verticillaris* | * |  |
| Rubiaceae | *Galium* | *Galium verum* | * |  |
| Rutaceae | *Haplophyllum* | *Haplophyllum dauricum* | * | * |
| Thymelaeaceae | *Stellera* | *Stellera chamaejasme* | * |  |

Note: * indicate species was recorded in the Inner Mongolia Grassland Ecosystem Research Station (ERS) and Xilinhot National Climate Observatory (NCO).

**REFERENCE**

Swenson, N.G., Enquist, B.J., Jill, T. & Zimmerman, J.K. (2007). The influence of spatial and size scale on phylogenetic relatedness in tropical forest communities. *Ecology*, 88, 1770-1780.

Webb, C.O., Ackerly, D.D., Mcpeek, M.A. & Donoghue, M.J. (2002). Phylogenies and Community Ecology. *Annual Review of Ecology and Systematics*, 8, 475-505.
